# Massage Therapy for Osteoarthritis of the Knee: A Randomized Dose-Finding Trial

**DOI:** 10.1371/journal.pone.0030248

**Published:** 2012-02-08

**Authors:** Adam I. Perlman, Ather Ali, Valentine Yanchou Njike, David Hom, Anna Davidi, Susan Gould-Fogerite, Carl Milak, David L. Katz

**Affiliations:** 1 Duke Integrative Medicine, Duke University School of Medicine, Durham, North Carolina, United States of America; 2 Prevention Research Center, Yale University School of Medicine, Derby, Connecticut, United States of America; 3 Clinical Trials Unit, Section of Infectious Diseases, Boston Medical Center, Boston, Massachusetts, United States of America; 4 School of Health Related Professions, University of Medicine and Dentistry of New Jersey, Newark, New Jersey, United States of America; Marienhospital Herne - University of Bochum, Germany

## Abstract

**Background:**

In a previous trial of massage for osteoarthritis (OA) of the knee, we demonstrated feasibility, safety and possible efficacy, with benefits that persisted at least 8 weeks beyond treatment termination.

**Methods:**

We performed a RCT to identify the optimal dose of massage within an 8-week treatment regimen and to further examine durability of response. Participants were 125 adults with OA of the knee, randomized to one of four 8-week regimens of a standardized Swedish massage regimen (30 or 60 min weekly or biweekly) or to a Usual Care control. Outcomes included the Western Ontario and McMaster Universities Arthritis Index (WOMAC), visual analog pain scale, range of motion, and time to walk 50 feet, assessed at baseline, 8-, 16-, and 24-weeks.

**Results:**

WOMAC Global scores improved significantly (24.0 points, 95% CI ranged from 15.3–32.7) in the 60-minute massage groups compared to Usual Care (6.3 points, 95% CI 0.1–12.8) at the primary endpoint of 8-weeks. WOMAC subscales of pain and functionality, as well as the visual analog pain scale also demonstrated significant improvements in the 60-minute doses compared to usual care. No significant differences were seen in range of motion at 8-weeks, and no significant effects were seen in any outcome measure at 24-weeks compared to usual care. A dose-response curve based on WOMAC Global scores shows increasing effect with greater total time of massage, but with a plateau at the 60-minute/week dose.

**Conclusion:**

Given the superior convenience of a once-weekly protocol, cost savings, and consistency with a typical real-world massage protocol, the 60-minute once weekly dose was determined to be optimal, establishing a standard for future trials.

**Trial Registration:**

ClinicalTrials.gov NCT00970008

## Introduction

Osteoarthritis (OA) is a slowly progressive degenerative disease of the joints that at present afflicts approximately 27 million Americans [1], [2]. With the aging of the “baby boom” population and increasing rates of obesity, the prevalence of OA is estimated to increase 40% by 2025 [3]. Conventional therapies for OA have limited effectiveness, and toxicities associated with suitable drugs thus often limit utilization, leaving many facing surgery or chronic, often debilitating, pain, muscle weakness, lack of stamina, and loss of function [3], [4], [5], [6], [7], [8], [9], [10]. In 2005, US costs from OA related absenteeism alone were estimated at $10.3 billion, and in 2007, OA increased aggregate annual medical care expenditures by $185.5 billion (in 2007 dollars) [11]. Well-publicized events such as the multiple lawsuits associated with rofecoxib and potential cardiac toxicity, as well as the removal of additional COX-2 inhibitors from the market, have lessened the public's confidence in pharmaceuticals and led to increased interest in therapeutic interventions believed to be safer [12], [13], [14].

Massage therapy and certain other complementary and alternative medicine (CAM) interventions are being utilized by OA sufferers, and represent attractive, potentially effective options to manage pain [12], [13], [14], [15], [16], [17], [18]. Massage is one of the most popular CAM therapies in the US [14]. Between 2002 and 2007, the 1-year prevalence of use of massage by the US adult population increased from 5% (10.05 million) to 8.3% (18.07 million) [14]. Massage is generally used to relieve pain from musculoskeletal disorders [19], [20], [21], [22], cancer, and other conditions; rehabilitate sports injuries; reduce stress; increase relaxation; decrease feelings of anxiety and depression; and aid general wellness [12], [21], [23], [24], [25], [26], [27], [28], [29], [30], [31], [32], [33], [34], [35]. However, only a relatively small body of research exists exploring the efficacy of massage therapy for any condition.

In 2006, we reported results of a pilot study of massage therapy for OA of the knee [18]. Subjects with OA of the knee meeting American College of Rheumatology Criteria [36] were randomized to biweekly (4 weeks), then weekly (4 weeks) Swedish massage (1 hour sessions) or wait list. Subjects receiving massage therapy demonstrated significant improvements in the Western Ontario and McMaster Universities Osteoarthritis Index (WOMAC) [37], [38], [39] pain, stiffness, and physical functional disability domains (p<0.001) and visual analog pain scale [39] (p<0.01) [18], compared to usual care. Notably, the benefits persisted up to 8 weeks following the cessation of massage [40]. Despite these promising results, there was no data to determine whether the dose utilized in the pilot study was optimal.

Here, we report the results of our Phase 2 dose-finding study to identify a dose and treatment regimen of an 8-week course of a standardized Swedish massage therapy for OA of the knee that is both optimal (providing greatest effectiveness) and practical (minimizing patient cost and inconvenience). To our knowledge, this was the first dose-finding study of massage therapy for OA, and for OA of the knee specifically. This trial employed a more diverse subject population at two clinical sites, and assessed longer-term residual effects, than did our earlier pilot study. In addition, a formally manualized massage protocol was developed and utilized [18]. Results of the trial reported herein will inform the dosing regimen for future clinical trials designed to confirm the efficacy of massage therapy for osteoarthritis of the knee and define its place in clinical practice.

## Methods

The protocol for this trial and supporting CONSORT checklist are available as supporting information; see Checklist S1 and Protocol S1.

### Ethics Statement

The study protocol, consent form and all recruitment materials were approved by the Institutional Review Boards of the University of Medicine and Dentistry of New Jersey (Newark, NJ), Griffin Hospital (Derby, CT), and the Saint Barnabas Medical Center (Livingston, NJ). The study was conducted in accordance with the Declaration of Helsinki.

### Participants

Eligible patients were men and women with radiographically-established OA of the knee who met American College of Rheumatology criteria [36], were at least 35 years of age, and had a pre-randomization score of 40 to 90 on the visual analog pain scale. Patients with bilateral knee involvement had the more severely affected knee (determined by the patient) designated as the study knee. Subjects using NSAIDS or other medications to control pain were included if their doses remained stable three months prior to starting the intervention.

Subjects were excluded if they suffered from rheumatoid arthritis, fibromyalgia, recurrent or active pseudogout, cancer, or other serious medical conditions. Subjects were also excluded if they had signs or history of kidney or liver failure; unstable asthma; knee replacement of both knees; reported recent use (4 weeks–1 year prior to enrollment) of oral or intra-articular corticosteroids or intra-articular hyaluronate; or knee arthroscopy or significant knee injury one year prior to enrollment. A rash or open wound over the knee and regular use of massage therapy (greater than once a month) also resulted in exclusion from the study.

**Figure 1:**
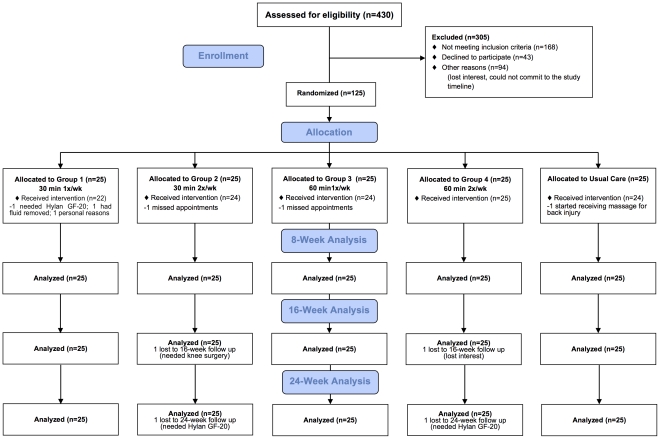
Participant Flow Diagram.

Those passing telephone screening (*n* = 125), agreeing to the study protocol, and providing a written physician confirmation of OA were scheduled for an on-site evaluation to provide written informed consent and undergo clinical eligibility screening. All subjects received nominal compensation (consisting of gift certificates for future massages) for their participation. The intervention was delivered at two sites; St. Barnabas Ambulatory Care Center (Livingston, NJ), and the Integrative Medicine Center at Griffin Hospital (Derby, CT). Subject participation and flow are shown in Figure 1.

Recruitment and screening commenced in July 2009 in Livingston, New Jersey and Southern Connecticut through flyers, newspaper advertisements, and press releases. Information letters were sent to patients identified with OA, as well as physicians in the rheumatology and internal medicine practices at Saint Barnabas Medical Center (Livingston, NJ) and Griffin Hospital (Derby, CT). Of the 430 persons screened for eligibility, 168 did not meet eligibility criteria, 43 refused to participate, and 94 were not screened for other reasons (Figure 1). The most common reasons for ineligibility were 1) no documented confirmation of OA of the knee, 2) recent history of cortisone and hyaluronate injections, and 3) history of knee replacement. Enrollment continued until July 2010. 125 subjects were randomized to receive one of four active massage arms (Groups 1–4) and one Usual Care/control arm.

### Randomization

Participants were block randomized using a permuted block design (blocks of 5 or 10) in a 1∶1∶1∶1∶1 ratio and stratified by site and body mass index (BMI) to ensure balance between the intervention and Usual Care groups across the two performance sites.. The study statistician (VYN) generated the random allocation sequence using SAS Software version 9.1. Each eligible and consented subject (identified by number) was assigned to one of the treatment arms. Treatment allocation was only known by the statistician and the study assistants (CM and AD) that assigned participants to interventions and generated visit schedules.

### Study Design and Interventions

This study was a randomized clinical trial with four treatment arms (and one Usual Care arm) designed to assess the effects of 8 weeks of a standardized Swedish massage protocol (see *Manualization*) provided at four distinct doses (Figure 1). Group 1 received 30 minutes of massage weekly for eight weeks. Group 2 received 30 minutes of massage twice weekly for the initial four weeks, and once weekly for the remaining four weeks. Group 3 received 60 minutes of massage weekly for eight weeks. Group 4 received 60 minutes of massage twice weekly for the initial four weeks, and once weekly for the remaining four weeks. Group 4 received the same dosing regimen as was given in our pilot trial [41]. The Usual Care group continued with their current treatment without the addition of massage therapy.

The doses were chosen as practical regimens that are commonly used in massage therapy and were designed to investigate the variables of length of individual treatment (30 min vs. 60 min), frequency (weekly vs. twice weekly), and total treatment time (240 min (Group 1, 30 min weekly×8 wk), 360 min (Group 2, 30 min biweekly×4 wk+30 min weekly×4 wk), 480 min (Group 3, 60 min weekly×8 wk), or 720 min (Group 4, 60 min biweekly×4 wk+60 min weekly×4 wk)).

### Manualization

Prior to enrolling subjects, a formal manualization process produced a study protocol that was tailored to subjects with OA of the knee, while respectful of the individualized nature of massage therapy [42]. The manualization process, conducted over the course of two months, involved the input of licensed massage therapists that participated in the pilot study, massage researchers, as well as the investigative team. The resulting massage protocol was made reproducible, using standard massage techniques [18], [43], as well as flexible for individual subject variability.

The manualized protocol specifies the body regions to be addressed; with distinct 30 and 60-minute protocols (Table 1), as well as the standard Swedish strokes to be used (effleurage, petrissage, tapotement, vibration, friction, and skin rolling) [18]. Each protocol specifies the time allocated to various body regions (lower/upper limbs, lower/upper back, head, neck, chest). The protocol was specifically designed to address symptoms of osteoarthritis of the knee. The order of body regions is not specified in order to account for individual practitioner preference. The manual specifies intentions/attentions for the study massage therapists. Each study therapist was taught and agreed to the protocol and signed a form attesting to adherence to the study protocol after each massage session. Study personnel reviewed adherence to the protocol at regular intervals throughout the study period. No deviations from the manualized massage protocol occurred.

**Table 1:** 30- and 60-Minute Massage Protocols.

30 minute protocol (25 minutes of table time)		
Region	Time Allotted	Distribution
Lower Limbs	12–15 min (45–50% of session)	From knee down including lower leg, ankle, and foot. From knee up including hips, pelvis, buttocks & thigh.
Upper Body	8–12 min (36–44% of session)	Lower and upper back. Head/Neck/Chest
Discretionary	2–5 min (6–19% of session)	Therapist to expand treatment to other affected areas; i.e. rib cage, flank, upper limbs, etc.

60 minute protocol (55 minutes of table time*)		
Lower Limbs	20–27.5 min (45–50% of session)	From knee down including lower leg, ankle, and foot. From knee up including hips, pelvis, buttocks and thigh.
Upper Body	15–24 min (36–44% of session)	Lower and upper back. Head, neck, and chest.
Discretionary	3.5–20 min (6–19% of session)	Therapist to expand treatment to other affected areas; i.e. rib cage, flank, upper limbs, etc.

*Accounting for time spent in transition including the welcome, transition to the massage room, taking off jewelry, and other preparatory activities.

### Primary outcome measure

The primary outcome measure was change in the Western Ontario and McMaster Universities Arthritis Index (WOMAC–Global). The WOMAC has been used extensively in the quantitative assessment of OA of the knee, and has been proven to be effective in assessing pain in those suffering from the illness [37], [38], [44]. It assesses dimensions of pain, functionality and joint stiffness through 24 questions. The WOMAC has been subject to numerous validation studies to assess reliability and responsiveness to change in therapy, including physical forms of therapy [37], [38], [44] and was successfully utilized in the Phase 1 study [18].

### Secondary outcome measures

Secondary measures included pain as measured on a visual analog scale (0 to 100 scale), a validated [39] mechanical scale used to measure pain sensation intensity evoked by nociceptive stimuli [45]. Other secondary outcomes were joint flexibility, defined as the range of motion (ROM) allowed at the knee during flexion using a double-armed goniometer, and measured time to walk 50 feet (15 m) on a level surface within the clinic facilities.

Outcomes were assessed at baseline, 8-weeks, 16-weeks, and 24-weeks post-baseline. Assessment visits were scheduled by a research assistant, though the measurements were assessed by separate personnel blinded to treatment assignments. Adherence was assessed by tracking the number of massage visits that subjects completed.

### Statistical Analysis

Statistical analysis was conducted using SAS software (Version 9.1, SAS Institute, Cary, NC). Repeated measures ANOVA using linear mixed model regression was used to determine between-group changes in all outcome measures and changes in domain-specific (i.e. pain, stiffness, and functionality) WOMAC measures, controlling for time-dependent variables. In all analyses, a two-tailed α of less than 0.05 was considered statistically significant. Duncan's multiple range test (a multiple comparison test) was used to determine whether means differ significantly across treatment groups. A dose-response curve was plotted assessing the magnitude of improvement on 8-week WOMAC Global scores plotted against the total 8-week dose of massage. A sample size of 125 individuals in five arms was based on budgetary and logistical constraints.

## Results

Of the 125 enrolled subjects, 119 completed the 8-week assessment and 115 completed the entire trial (Figure 1). Subjects received the intervention between November 2009 and October 2010.

Baseline characteristics of the study participants are provided in Table 2. The randomization process with stratification by BMI was largely successful in producing equivalent groups. The only differences seen at baseline were that Group 1 was older than the other groups, and Group 3 had more perceived pain than usual care as assessed by the visual analog scale, though no differences were seen in the pain subscale of the WOMAC.

**Table 2:** Demographic Characteristics and Baseline Values.

Variable	Group 1 (n = 25)	Group 2 (n = 25)	Group 3 (n = 25)	Group 4 (n = 25)	Usual Care (n = 25)
Gender					
Female	15 (12.0%)	18 (14.4%)	19 (15.2%)	17 (13.6%)	19 (15.2%)
Male	10 (8.0%)	7 (5.6%)	6 (4.8%)	8 (6.4%)	6 (4.8%)
Race					
White	23 (18.4%)	22 (17.6%)	19 (15.2%)	20 (16.0%)	22 (17.6%)
Asian	0 (0.0%)	0 (0.0%)	0 (0.0%)	1 (0.8%)	0 (0.0%)
White/Asian	0 (0.0%)	1 (0.8%)	0 (0.0%)	0 (0.0%)	0 (0.0%)
African American	2 (1.6%)	2 (1.6%)	4 (3.2%)	4 (3.2%)	2 (1.6%)
Hispanic	0 (0.0%)	0 (0.0%)	1 (0.8%)	0 (0.0%)	0 (0.0%)
Unknown	0 (0.0%)	0 (0.0%)	1 (0.8)	0 (0.0%)	1 (0.8%)
Age (years)	69.9±8.6	61.9±9.5	62.6±10.6	63.6±13.0	63.6±10.2
Body Mass Index (kg/m^2^)	31.0±7.5	32.1±6.8	31.8±6.7	31.3±7.1	31.7±6.5
WOMAC (mm)					
Pain	52.3±19.9	42.4±23.0	52.5±16.5	44.4±19.3	46.3±15.4
Stiffness	53.4±24.1	58.6±21.1	58.4±24.7	51.2±24.4	62.8±18.2
Functionality	52.9±17.9	49.5±19.5	49.8±19.7	48.3±20.2	50.5±17.4
Global	52.9±18.3	50.2±19.4	53.6±17.3	48.0±19.0	53.2±14.8
Pain (VAS) (mm)	61.2±16.8	64.0±12.7	66.4±11.3	59.2±13.3	57.6±9.0
50-foot walk (seconds)	18.3±6.9	16.8±7.0	15.6±3.2	15.7±5.4	15.7±2.8
Range of Motion (degrees)	108.7±14.6	114.4±10.4	115.3±10.5	112.8±12.6	115.6±9.6

Values are mean ± SD except otherwise stated.

### Primary Outcome

WOMAC Global scores improved significantly (24.0 points, 95% CI ranged from 15.3–32.7) in the 60-minute massage groups compared to Usual Care (6.3 points, 95% CI 0.1–12.8) at the primary endpoint of 8-weeks (Table 3). No statistically significant differences between the massage groups were detected at 8 weeks, though the magnitude of change in the groups receiving 60-minute doses (Groups 3 and 4) was greater than the magnitude of change in the groups receiving 30-minute doses (Groups 1 and 2) (Table 3).

**Table 3:** Mean change (95% CI) in outcomes at 8-weeks post-baseline (primary endpoint).

GROUP (n)	Group 1 (n = 22)	Group 2 (n = 24)	Group 3 (n = 24)	Group 4 (n = 25)	Usual Care (n = 24)
DOSE	30 min 1×/wk	30 min 2×/wk	60 min 1×/wk	60 min 2×/wk	(no massage)
TOTAL MASSAGE RECEIVED	240 min	360 min	480 min	720 min	0 min
WOMAC (mm)					
Pain	−15.1 (−23.4, −6.8)	−14.4 (−23.8, −5.1)	−27.2 (−36.3, −18.0)†	−27.7 (−36.9, −18.6)†	−5.6 (−13.1, 1.9)
Stiffness	−19.0 (−30.4, −7.6)	−23.4 (−34.5, −12.3)	−23.7 (−34.6, −12.7)	−22.3 (−32.9, −11.6)	−6.7 (−15.7, 2.2)
Functionality	−18.0 (−25.5, −10.4)	−17.2 (−26.9, −7.6)	−21.2 (−29.3, −13.1)†	−22.0 (−31.6, −12.5)†	−6.6 (−12.2, −0.9)
Global	−17.4 (−25.3, −9.4)	−18.4 (−27.5, −9.2)	−24.0 (−32.1, −15.9)†	−24.0 (−32.7, −15.3)†	−6.3 (−12.8, 0.1)
Visual Analog Scale (mm)	−14.2 (−25.0, −3.4)	−26.1 (−36.8, −15.3)	−39.8 (−48.1, −31.4)† ‡	−31.2 (−39.4, −22.9)†	−9.8 (−18.6, −1.1)
50-foot walk (seconds)	−1.3 (−3.0, 0.4)	−2.4 (−4.7, −0.1)	−1.7 (−2.7, −0.6)	−2.0 (−3.4, −0.6)	−1.3 (−2.4, −0.2)
Range of Motion (degrees)	−2.5 (−7.5, 2.6)	−4.8 (−9.9, 0.4)	−1.3 (−6.2, 3.5)	−6.6 (−11.6, −1.6)	0.2 (−4.1, 4.4)

Values are mean with 95% confidence intervals; negative values indicate improvement;

† Significant (non-overlap) compared to Usual Care;

‡ Significant (non-overlap) compared to Group 1.

### WOMAC Pain Subscale

The 60-minute doses (Groups 3 and 4) were also significantly (27.2–27.7 points, 95% CI ranged from 18.0–36.9 points) improved compared to usual care (5.6 points, 95% CI ranged from −1.9–13.1) at 8-weeks (Table 3). Significant improvement (p<0.05) from baseline was achieved for all massage groups at 8 weeks, and at 16 and 24 weeks for the three highest doses of massage (Groups 2, 3 and 4), whereas no improvement (p>0.05) in Usual Care was seen at any time point (Tables 3 and 4).

**Table 4:** Changes in outcomes at 24-weeks post-baseline.

GROUP (n)	Group 1 (n = 22)	Group 2 (n = 24)	Group 3 (n = 24)	Group 4 (n = 25)	Usual Care (n = 24)
DOSE	30 min 1×/wk	30 min 2×/wk	60 min 1×/wk	60 min 2×/wk	(no massage)
WOMAC (mm)					
Pain	−12.2 (−22.4, −2.0)	−3.9 (−12.7, 4.9)	−13.7 (−23.4, −4.0)	−14.2 (−24.5, −3.8)	−7.5 (−16.0, 1.1)
Stiffness	−15.4 (−26.4, −4.5)	−9.6 (−20.6, 1.3)	−16.9 (−28.5, −5.2)	−16.8 (−29.7, −3.9)	−6.4 (−13.2, 0.4)
Functionality	−15.3 (−24.5, −6.1)	−7.4 (−14.8, 0)	−12.1 (−22.0, −2.1)	−14.4 (−23.4, −5.4)	−4.2 (−11.1, 2.7)
Global	−14.3 (−22.9, −5.7)	−7.0 (−15.6, 1.6)	−14.2 (−23.4, −5.0)	−15.1 (−25.1, −5.1)	−6.0 (−12.6, 0.5)
Visual Analog Scale (mm)	−14.4 (−25.9, −2.8)	−14.0 (−24.7, −3.3)	−18.5 (−29.0, −8.1)	−22.8 (−35.5, −10.1)	−11.5 (−21.0, −2.0)
Time to walk 50 Feet (seconds)	−2.4 (−4.4, −0.4)	−1.8 (−3.6, 0)	−1.4 (−2.8, 0)	−1.8 (−3.3, −0.3)	−1.4 (−2.6, −0.2)
Range of Motion (degrees)	−4.7±(−10.9, 1.5)	0.4 (−5.7, 4.8)	−2.7 (−7.6, 2.3)	−8.3 (−14.2, −2.5)	−0.3 (−4.7, 4.1)

Values are mean with 95% confidence intervals; negative values indicate improvement.

### WOMAC Stiffness Subscale

No significant between-group changes were seen in WOMAC Stiffness, though the magnitude of change was greater in all treatment groups compared to usual care.

### WOMAC Functionality Subscale

The 60-minute doses (Groups 3 and 4) were significantly (21.2–22.0 points, 95% CI ranged from 12.5–31.6 points) improved compared to usual care (6.6 points, 95% CI ranged from 0.9–12.2) at 8-weeks (Table 3).

### Dose Response

**Figure 2:**
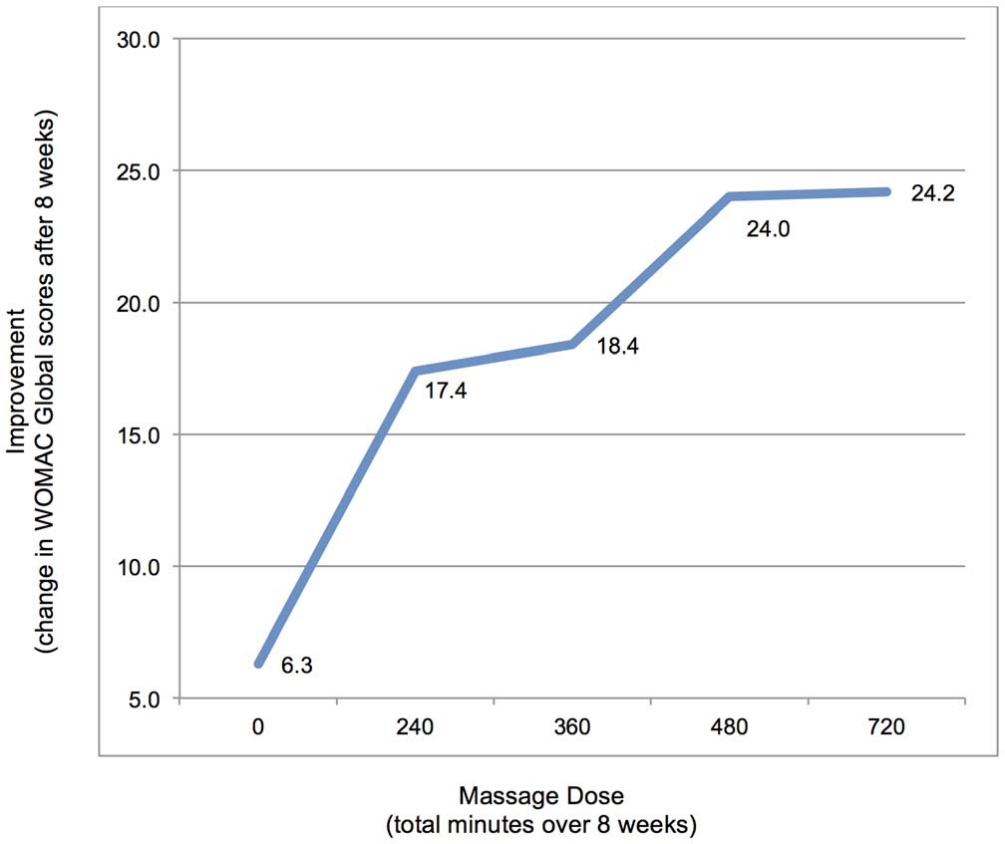
Dose-Response Curve. Dose-response curve plotting dose (total minutes over the course of 8-weeks of massage) (x-axis) vs. improvement (change in WOMAC Global scores after 8-weeks). Dose-response effects plateaued at 480-minutes (Group 3), with no significant improvements noted in the 720-minute (Group 4) dose.

A dose-response curve based on changes in WOMAC Global scores at 8-weeks shows increasing effect with greater total time of massage, but with a plateau at the 480-minute dose (Group 3) (Figure 2).

### Visual Analog Pain Scale

Pain perception improved significantly (31.2–39.8 points, 95% CI ranged from 22.9–48.1 points) in both 60-minute dose groups (Groups 3 and 4) compared to Usual Care at 8-weeks post baseline, with no significant differences between Groups 3 and 4. All treatment groups reported decreased pain perception compared to baseline at all time points (Tables 3 and 4).

### Timed 50-foot walk

All groups showed decreased time to walk 50 feet at all time points, except group 1 at week 8 (Tables 3 and 4). However, there were no significant differences between the groups at any timepoint.

### Range of Motion

No significant between-group differences were seen in range of motion at 8-weeks post-baseline, though all massage groups changed in the positive direction. Group 4, the highest total dose of massage, was significantly improved from baseline at all time points. The 30-minute protocols and Usual Care groups did not demonstrate any significant changes from baseline at any timepoint.

### Adherence

Subjects completing 80% or more of assigned visits (8 or 12 visits, based on treatment assignment) were considered adherent; 119/125 subjects were adherent to all assigned massage visits with no significant differences in adherence between treatment assignments (Figure 1).

### Adverse Effects

No adverse effects related to the intervention were seen during the course of the study.

## Discussion

This was the first formal dose-finding study of massage therapy for any condition. This study investigated four different doses of tailored Swedish massage, varying both the time (30 vs. 60 minutes per treatment) and frequency (once a week vs. twice a week for the first month) to determine an optimal, practical dose to use in future studies. Our manualized protocol incorporated standard Swedish massage techniques focused on osteoarthritis of the knee, based on the protocol used in our Phase 1 study. We operationally defined ‘optimal-practical’ as ‘producing the greatest ratio of desired effect compared to costs (in time, labor, and convenience).’ If the most effective dose were the least labor-intensive, ‘optimal’ and ‘optimal-practical’ would be the same.

We were also interested in seeing whether the promising results of our pilot study would be repeated and extended with this now manualized protocol of Swedish massage, with a more diverse population at two sites, with a longer follow up period. The potential effectiveness of this treatment was further supported in this study, as all massage doses demonstrated significant improvement from baseline, as well as differences from usual care in WOMAC Global scores at the termination of massage (8-week timepoint). In addition, all massage groups reported significantly decreased WOMAC Pain as assessed by VAS at the 8-week timepoint compared to baseline, which was also different from usual care for the three highest doses.

Angst et. al estimated minimal clinically important differences (MCID) in WOMAC global scores (for improvement) to be 18% change from baseline [46], while Escobar et al. found that a MCID is approximately 15 points in patients following total knee replacement [47]. In our study, subjects receiving the 60-minute doses improved a mean of 24.0 points in WOMAC global scores (44%–50% change from baseline). Thus, the magnitude of change in WOMAC scores is highly clinically significant.

Our results suggest a benefit of increasing massage dose with diminishing returns at the highest level. Our dose-response curve based on WOMAC Global scores indicates increasing improvement with greater total dose (minutes) of massage, with a threshold effect at the 480-minute dose (Group 3). While it is difficult to tease out differences due to total dose and effectiveness of 30 vs. 60 minute individual treatments, due to the design of the study, there is a clear trend for greater magnitude of changes in our outcomes in both of the 60-minute doses (Groups 3 and 4), compared to baseline, usual care, and the 30-minute dose groups. There were no significant differences when comparing the 60-minute groups to each other at any time point or for any outcome. WOMAC subscales generally followed the pattern of the global scores, with strongest responses for the higher doses.

The durability of the response in improvement of OA symptoms and functionality to massage treatment was also supported by the results of this study. Our Phase 1 trial [18] demonstrated significant effects of massage therapy 16-weeks post-baseline on the WOMAC, visual analog pain scale, and the 50-foot timed walk. Anticipating similar effects, we assessed subjects at 16 weeks and extended our observations to 24-weeks post-baseline. Although the magnitude of effects was strongest after 8 weeks of treatment and generally decreased with time, the persistence of improvements at 2 months and 4 months after treatment cessation indicate that the effects of the 8 weeks of massage go beyond immediate changes to longer term shifts that may be global and/or localized to the knee, suggesting that periodic maintenance doses of massage may help sustain effects over time. The mechanisms for such persistent benefit are not fully elucidated, and clearly warrant investigation.

**Figure 3:**
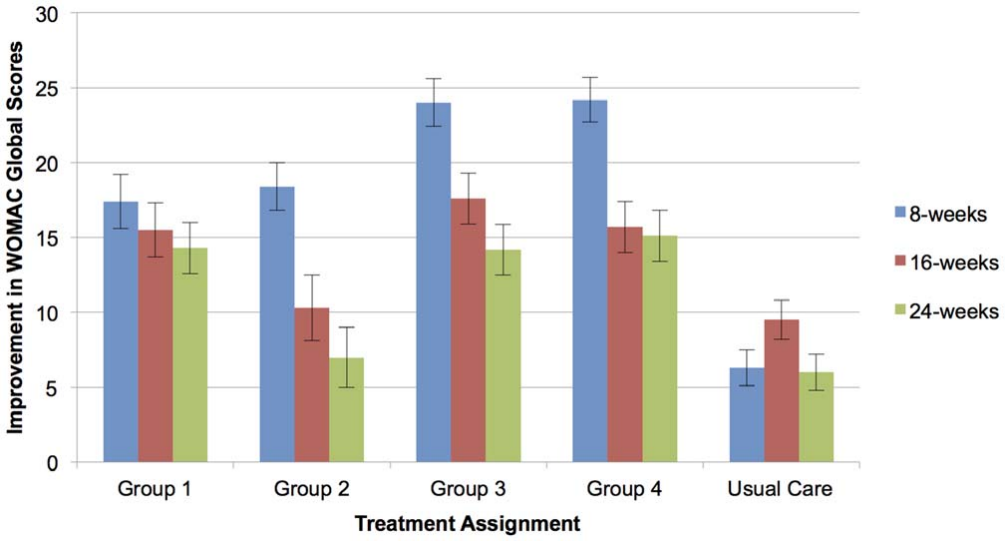
Improvement in WOMAC-Global Scores at Assigned Doses of Massage.

All massage groups demonstrated significant improvement in WOMAC Global scores at 16 and 24 week timepoints compared to baseline, whereas those in the usual care group did not (Figure 3). The three highest doses of massage improved relative to baseline in WOMAC pain at 16 and 24 weeks, in stiffness at 24 weeks, and functionality at 16 and 24 weeks.

All groups, including usual care, demonstrated decreases in the timed 50-foot walk compared to baseline, but no clear patterns of dose effect or significant differences between the groups. Larger groups and perhaps a 100-foot walk may be needed to detect between-group differences. Although almost no statistically significant differences between the massage groups and Usual Care were seen in the 16 and 24 week time points, the directionality of all changes was towards improvement, and the magnitude of changes seen was greater than changes seen in Usual Care (Table 4). Our sample size was inadequate to determine statistically significant between-group changes, as the goal for this Phase 2 trial was to determine an optimal-practical dose, rather than to determine efficacy.

Future studies should incorporate larger samples that adequately power for between group changes in longer term effects of massage therapy. The improvements in the Usual Care arm in some outcome measures, possibly due to Hawthorne [48] or other nonspecific effects [49], reduced the magnitude of between-group differences, and reaffirm the importance of control groups in this type of study.

Massage is theorized to work through a variety of mechanisms. Increased blood circulation to the muscles promoting gas exchange and delivery of nutrients and removal of waste products has long been thought to be one of the outcomes and benefits of massage, and recent studies support this effect [50], [51], [52], [53]. There is some evidence for the promotion of a relaxation response and shift to parasympathetic nervous system activation, with reduced heart rate, blood pressure, biochemical, (including blood and salivary stress hormones, endorphins, and serotonin), and brain activation changes, associated with reduced anxiety [40], [54], [55], [56], [57], [58], [59]. This may be mediated through the activation of mechanoreceptors in the deep tissues innervated by alpha beta fibers with subsequent central nervous system (CNS) effects on the pituitary gland and limbic system and/or other mechanisms [60]. The need for moderate pressure to achieve many of the effects of massage therapy may support this mechanism, deserves further investigation, and supports light touch as an appropriate active control for future trials [25], [51], [55], [58], [61], [62]. A recent study comparing a single session of Swedish massage to light touch showed significant neuroendocrine and immune system changes over time, with differing patterns and degree in the massage and control intervention [59]. Other potential outcomes and mechanisms of massage therapy's effectiveness include decreasing muscle strain, balancing muscle tension across the joint, positive mechanical changes in muscles, increased joint flexibility and proprioception, increased lymphatic circulation, immunologic and inflammatory changes, improved sleep, and blocking pain signals [32], [53], [63], [64], [65], [66], [67]. Research dedicated to exploring the mechanisms of effect is clearly warranted.

Limitations of this trial included a small sample. Further, the truly “optimal” dose might differ from any of the four studied. Although additional doses for the massage intervention could have been considered, the regimens that were assessed conform well to massage regimens currently in use, are advocated by massage therapists, and were practical to implement. Our trial was also limited to Swedish massage techniques. This limits generalizability to other techniques, but offers the advantage of clear standardization of the intervention. Swedish technique predominates in clinical settings [68] and is thus the logical, initial choice; other massage techniques should be compared to Swedish massage once its efficacy is established. Further, the Swedish technique has been successfully applied and demonstrated promise in our pilot study [18].

As the mechanism(s) of action of massage are not fully elucidated, it is premature to predict dose-responses for higher or lower doses than the doses utilized in this trial, or applied to clinical models besides osteoarthritis of the knee. For example, if massage enhances regional blood flow, it might be that a follow-up massage too soon (i.e. within one week) actually attenuates benefit by applying pressure to slightly engorged tissue. Thus, there might be an optimal periodicity to massage, with suboptimal effects seen with dosing outside this purported ideal. Changes in neuroendocrine and inflammatory status, pain generation and sensitivity, or musculature strain or balance, may also reach an optimal state, which persists for some time, and is not enhanced by further massage within a weeks' time. Our study, however, did not attempt to establish a dose-response gradient. Rather, the goal was to establish an optimal-practical dose for future testing, based on the combination of convenience, practicability, and therapeutic effectiveness.

### Conclusion

This dose-finding trial established an optimal dose of 60-minutes of this manualized Swedish massage therapy treatment delivered once weekly in an eight week protocol for OA of the knee. This decision was based on the superiority of the 60 minute compared to 30 minute treatments, the essentially equivalent outcomes of the two 60 minute doses, the convenience of a once-weekly protocol (compared to biweekly), cost savings, and consistency with a typical real-world massage protocol. As there is promising [18], but not definite [69], potential for the utility of massage therapy for knee osteoarthritis, future research on this standardized approach to massage therapy should utilize this dose. In addition, future, more definitive research is needed investigating not only the efficacy, but also cost-effectiveness of massage for OA of the knee and other joints, as well as research exploring the mechanism(s) by which massage may exert its effects in this clinical application and in general.

## Supporting Information

Checklist S1 **CONSORT Checklist.**
(DOC)

Protocol S1 **Trial Protocol.**
(PDF)
