# Supplementary material for: Massage Therapy for Osteoarthritis of the Knee: A Randomized Dose-Finding Trial
Source: PLoS One. 2012 Feb 8;7(2):e30248. doi: 10.1371/journal.pone.0030248 (PMC3275589; doi:10.1371/journal.pone.0030248)
Supplement: Protocol S1 — Trial Protocol. (PDF) [file pone.0030248.s002.pdf]

UMDNJ
UNIVERSITY OF MEDICINE & DENTISTRY OF NEW JERSEY

Institutional Review Board
Newark Campus

NOTICE OF APPROVAL OF MODIFICATION

IRB PROTOCOL NUMBER: 0120090141
(Refer to this number when making inquiries)

PRINCIPAL INVESTIGATOR/DEPT: **Adam Perlman, M.D., M.P.H., F.A.C.P.**
Interdisciplinary Studies
School of Health Related Professions
Stanley S. Bergen Building
65 Bergen Street 101
Newark, NJ 07101

CO-INVESTIGATOR(S): David Hom, M.S.

TITLE: Exploring Massage Benefits for Arthritis of the Knee (EMBARK)

PERFORMANCE SITE(S): UMDNJ- Stanley S. Bergen Building
St. Barnabas Ambulatory Care Center- Livingston, NJ 07039
Yale-Griffin Prevention Research Center- Derby, CT 06418

SPONSOR/PROTOCOL NUMBER: Department Funded

TYPE OF REVIEW: Expedited

RISK DETERMINATION LEVEL: Minimal Risk

DEVICE DETERMINATION: Not Applicable

TYPE OF APPROVAL: MODIFICATION

MODIFICATION ITEMS: Study Personnel, Study Materials and Protocol

Document Versions Approved with this Modification:

☑ **Protocol Version:** Version 2.1 9/30/2009

☑ **Other items:** Flyer - Do You Have Knee Pain from Osteoarthritis? (12/18/09); Pain Mediciation Log (12/18/09); Adverse Event Log (12/18/09); Health and Medications Changes (12/18/09); Commitment Agreement (12/18/09); Statement of Gift Certificate receipt(s) -(a) for two 60 minute massage sessions (12/18/09) & (b) for six 30 minute massage sessions (12/18/09); Letter to subject's physician - Dear Dr. ___ (12/18/09); WOMAC Questionnaire (version 2.1, 9/30/09); Subject Intake Form (version 2.1, 9/9/09); Walk 50 Feet / ROM Form (version 2.1, 9/30/09)

Currently Approved Documents:

Protocol Version: Version 2.1- 9/30/2009
Consent Version: Version 2.1-8/21/2009
Other items: Flyer - Do You Have Knee Pain from Osteoarthritis? (12/18/09); Pain Mediciation Log (12/18/09); Adverse Event Log (12/18/09); Health and Medications Changes (12/18/09); Commitment Agreement (12/18/09); Statement of Gift Certificate receipt(s) -(a) for two 60 minute massage sessions (12/18/09) & (b) for six 30 minute massage sessions (12/18/09); Letter to subject's physician - Dear Dr. ___ (12/18/09); WOMAC Questionnaire (version 2.1, 9/30/09); Subject Intake Form (version 2.1, 9/9/09); Walk 50 Feet / ROM Form (version 2.1, 9/30/09) ; NIH Grant; Letters of approval from Griffin Health Services Corporation (9/30/09) and Saint Barnabas Health Care System (9/9/09); Clinicaltrials.gov Protocol Registration receipt (8/31/09)

ClinicalTrials.gov
Protocol Registration System

APPROVED
0120090141
DEC 18 2009
UMDNJ-NEWARK CAMPUS
INSTITUTIONAL REVIEW BOARD

Protocol Registration Receipt
08/31/2009

Exploring Massage Benefits for Arthritis of the Knee (EMBARK)

This study is not yet open for participant recruitment.

Verified by National Center for Complementary and Alternative Medicine (NCCAM), August 2009

Sponsored by:	National Center for Complementary and Alternative Medicine (NCCAM)
Information provided by:	National Center for Complementary and Alternative Medicine (NCCAM)
ClinicalTrials.gov Identifier:	NCT00970008

Purpose

In 2004, we conducted a pilot randomized, wait list controlled, trial (RCT) of massage therapy for OA of the knee in 68 subjects. That study, supported by CDC grant SIP-14-00, revealed the potential efficacy of Swedish massage therapy in the treatment of OA of the knee, with benefits of increased function and decreased pain persisting at least eight weeks following treatment cessation. The results of that trial, the first RCT of massage for OA, were published in the Archives of Internal Medicine in 2006 (See reference in More Information section). This current project builds on the design and findings of the pilot trial to determine the optimal dose and treatment regimen and provide longer term follow up. This project is a dual-site, randomized, dose-ranging trial to compare four dose/regimens in order to identify the optimal protocol for clinical practice.

The primary study hypothesis is that an eight (8) week course of Swedish massage therapy of one of the four proposed doses (by frequency and duration of massage treatment session) will be effective in reducing pain and improving function in patients with confirmed OA of the knee.

Condition	Intervention	Phase
Osteoarthritis of the Knee	Swedish Massage 30 min 2x/wk x4 wks then 1x/wk x4 wks<br>Swedish massage 60 min 2x/wk for 4 wks then 1x/wk for 4 wks	Phase 2

26887

EMBARK STUDY: WALK 50 FEET / ROM FORM

APPROVED
01200901 41
DEC 18 2009
UMDNJ-NEWARK CAMPUS
INSTITUTIONAL REVIEW BOARD

PATIENT ID #: ☐☐☐☐ VISIT#: ☐

DATE: ☐☐ / ☐☐ / ☐☐☐☐
MONTH DAY YEAR

STUDY JOINT

○ Right Knee ○ Left Knee

TIME TO WALK 50 FEET

________________ SECONDS

Staff/Investigator Name: ________________________ Initials: __________

Date: ☐☐ / ☐☐ / ☐☐☐☐

RANGE OF MOTION

________________ DEGREES

Staff/Investigator Name: ________________________ Initials: __________

Date: ☐☐ / ☐☐ / ☐☐☐☐

EMBARK STUDY – Subject Intake Form

Patient ID #: ☐ ☐ ☐ Site: ________ Referring Physician: ____________________

Age: ____ Ht: ____________ Wt: ____________ BMI: ______ ☐ Male ☐ Female

Past Medical History: ____

____

____

____

Medications / Supplements: ____

____

____

____

Allergies: ____

Inclusion Criteria:

☐Yes ☐No: Consent Signed __________ Date

☐Yes ☐No: Established diagnosis of OA meeting ACR criteria

☐Yes ☐No: 35 years of age or greater

☐Yes ☐No: A score of 4-9 on a visual analogue scale.

☐Yes ☐No: Stable on medications for 3 months

Exclusion criteria:

☐Yes ☐No: Diagnosis of RA

☐Yes ☐No: History of fibromyalgia, recurrent or active pseudogout, cancer or other serious disease, signs or history of kidney or liver failure.

☐Yes ☐No: Asthma requiring steroids, oral corticosteroids within the last 4 weeks.

☐Yes ☐No: Intra-articular knee depo-corticosteroids within the previous 3 months.

☐Yes ☐No: Intra-articular hyaluronate within the previous 6 months.

☐Yes ☐No: Arthroscopy of the knee within the previous year.

☐Yes ☐No: Knee replacement. ☐ Right ☐ Left

☐Yes ☐No: Significant injury to the knee within the previous 6 months.

☐Yes ☐No: Rash or open wound over the knees.

☐Yes ☐No: Currently receiving massage therapy at least twice per month.

Patient ID #: ☐ ☐ ☐

APPROVED
0120090141
DEC 18 2009
UMDNJ-NEWARK CAMPUS
INSTITUTIONAL REVIEW BOARD

31727

EMBARK STUDY: WOMAC QUESTIONNAIRE

APPROVED
0120090141
DEC 18 2009
UMDNJ-NEWARK CAMPUS
INSTITUTIONAL REVIEW BOARD

PATIENT ID #: ☐☐☐☐ VISIT#: ☐

DATE: ☐☐ / ☐☐ / ☐☐☐☐
MONTH DAY YEAR

The following questionnaire is to be completed by the patient in black ball point pen. Was the evaluation completed?

A ○ Yes
B ○ No (If no, give reason in comments)

____

____

____

Date Completed: ☐☐ / ☐☐ / ☐☐☐☐
MONTH DAY YEAR

Comments:__________________________________

Study Joint:

A ○ Left knee
B ○ Right knee

Staff/Investigator Name ____________ Initials ______ Date ________

SAINT BARNABAS HEALTH CARE SYSTEM

Saint Barnabas Ambulatory Care Center

APPROVED
01020090141
DEC 18 2009
UMDNJ-NEWARK CAMPUS
INSTITUTIONAL REVIEW BOARD

RONALD J. DEL MAURO
President and Chief Executive Officer

SUSAN GARRUBBO
Executive Director
Director of Medical Affairs
(973) 322-7281
Fax (973) 322-7283

Dear Dr. ______________________________:

Your patient, ______________________________, has consented to be involved in a NIH-funded study evaluating the effects of massage therapy in the treatment of osteoarthritis of the knee. The study is being conducted by Dr. Adam Perlman of the Siegler Center for Integrative Medicine, (973) 322-7007. In order to assure that this patient fits the inclusion criteria of having an established diagnosis of osteoarthritis meeting American College of Rheumatology (ACR) criteria, please check the appropriate box below, sign this form and kindly fax this letter back to Carl Milak at (973) 972-5572. Thank You.

☐ I confirm that this patient meets the ACR criteria for diagnosis of osteoarthritis of the knee.

☐ I cannot confirm that this patient meets the ACR criteria for diagnosis of osteoarthritis of the knee.

Name (please print): ______________________________

Signature: ______________________________ Date: ________________

I, ______________________________, give permission for my doctor, ________________, to release information regarding my diagnosis of osteoarthritis of the knee to the Siegler Center for Integrative Medicine at the Saint Barnabas Ambulatory Care Center for its use in a NIH-funded study evaluating the effects of massage therapy in the treatment of osteoarthritis of the knee. I understand this study is being conducted under the direction of the University of Medicine & Dentistry of New Jersey (UMDNJ). Please fax the completed form to Carl Milak at (973) 972-5572.

Name (please print): ______________________________

Signature: ______________________________ Date: ________________

STATEMENT OF GIFT CERTIFICATE RECEIPT

I, ________________________________ certify that I have received gift certificates for two (2) 60 minute massage sessions for my participation in the EMBARK research study. I understand that they are to be redeemed within one (1) year of the date of my final study visit, and may only be used at the Siegler Center for Integrative Medicine at the Saint Barnabas Ambulatory Care Center, Livingston, NJ.

Signature ________________________

Date ___________________________

APPROVED
0120090141
DEC 18 2009
UMDNJ-NEWARK CAMPUS
INSTITUTIONAL REVIEW BOARD

STATEMENT OF GIFT CERTIFICATE RECEIPT

I, ________________________________ certify that I have received gift certificates for six (6) 30 minute massage sessions for my participation in the EMBARK research study. I understand that they are to be redeemed within one (1) year of the date of my final study visit, and may only be used at the Siegler Center for Integrative Medicine at the Saint Barnabas Ambulatory Care Center, Livingston, NJ.

Signature ________________________

Date ____________________________

APPROVED
0120090141
DEC 18 2009
UMDNJ-NEWARK CAMPUS
INSTITUTIONAL REVIEW BOARD

APPROVED
01-2009-0141
DEC 18 2009
UMDNJ-NEWARK CAMPUS
INSTITUTIONAL REVIEW BOARD

EMBARK STUDY

UMDNJ

COMMITMENT AGREEMENT

Participant Name______________________________ Date______________

Upon enrollment into this study, you will need to be able to make the following commitments.

Please initial each paragraph indicating you have read and agree to the commitment.

Initials

_____ I understand this study is designed to compare the effects of various amounts of massage therapy on symptoms of osteoarthritis of the knee. The effect is evaluated by written questionnaires and measuring your ability to move your joints and walk 50 feet. These tests are non-invasive.

_____ I understand that I need to have radiographically (x-ray) confirmed diagnosis of osteorarthritis of the knee in order to participate in the study. My primary care physician needs to sign a note confirming this. I am responsible for obtaining the signed note from my physician's office. In addition, I will complete the required paperwork including the consent form.

_____ I understand that the study is scheduled to begin approximately in September 2009 and my involvement will last for approximately six months. I understand that I will be randomly assigned to one of five different treatment groups (4 various doses of massage or usual care) for eight weeks. Additionally, I understand that I will have to return for assessment two times after the completion of the initial eight weeks: at 16 weeks and 24 weeks. I understand that I must come to the Siegler Center at the Saint Barnabas Ambulatory Care Center to have these tests completed. These appointments will be scheduled during business hours, Monday – Friday, between the hours of 8:00am – 5:00pm.

_____ I understand that the researchers involved in this study will make a reasonable attempt to accommodate scheduling requests, however, I understand that may not always be possible. Therefore, as a participant in this study, I agree to comply with the schedule and appointment times that are assigned to me.

_____ I understand that I will receive gift certificates for six (6) 30-minute sessions of massage therapy for my participation in this study if I was assigned to the usual care group, or gift certificates for two (2) 60 minute massages if I was assigned to one of the massage intervention groups. These are only valid for use at the Saint Barnabas Ambulatory Care Center, and must be used within one year of issue. The gift certificates will be given to me at the completion of the 16 week and 24 week follow-up assessments.

Please note any special requests or preferences you may have.

____

____

If, for any reason, you feel that you will not be able to comply with any of the above commitments, please notify our research staff.

EMBARK STUDY: PAIN MEDICATION LOG

Subject Number: ____________________

PLEASE RECORD THE USE OF ALL PRESCRIPTION/NON-PRESCRIPTION PAIN MEDICATION THAT YOU TOOK DURING THE MONTH ON AN "AS-NEEDED" BASIS. DAILY PAIN MEDICATION CAN BE RECORDED ONCE/MONTH.

Date	Medication	Dose	Time	Comments

Received by: ____________________ Date: ________ (final approval of completed log)

APPROVED
0120090141
DEC 18 2009
UMDNJ-NEWARK CAMPUS
INSTITUTIONAL REVIEW BOARD

EMBARK STUDY – ADVERSE EVENT LOG
(TO RECORD ANY ADVERSE EVENTS ON STUDY KNEE RELATED TO THE MASSAGE INTERVENTION)

Subject Number: ____________

Which knee (left/right?)	Describe the problem	Severity	Massage related?	Start Date/Time	Stop Date/Time
		☐ Mild<br>☐ Moderate<br>☐ Severe<br>☐ Life-threatening	☐Unrelated<br>☐Possible<br>☐Probably<br>☐Definitely	Date:<br>Time:	Date:<br>Time:
		☐ Mild<br>☐ Moderate<br>☐ Severe<br>☐ Life-threatening	☐Unrelated<br>☐Possible<br>☐Probably<br>☐Definitely	Date:<br>Time:	Date:<br>Time:
		☐ Mild<br>☐ Moderate<br>☐ Severe<br>☐ Life-threatening	☐Unrelated<br>☐Possible<br>☐Probably<br>☐Definitely	Date:<br>Time:	Date:<br>Time:
		☐ Mild<br>☐ Moderate<br>☐ Severe<br>☐ Life-threatening	☐Unrelated<br>☐Possible<br>☐Probably<br>☐Definitely	Date:<br>Time:	Date:<br>Time:
		☐ Mild<br>☐ Moderate<br>☐ Severe<br>☐ Life-threatening	☐Unrelated<br>☐Possible<br>☐Probably<br>☐Definitely	Date:<br>Time:	Date:<br>Time:

Received by: ______________________________ Date: ________ (final approval of completed log)

APPROVED
0120090141
DEC 18 2009
UMDNJ-NEWARK CAMPUS
INSTITUTIONAL REVIEW BOARD

EMBARK STUDY – HEALTH AND MEDICATION CHANGES
(TO RECORD OVERALL AND MAJOR HEALTH/MEDICATION CHANGES)

Subject Number: ____________

Date / Time	Describe the medical problem	Change in medication use?	Dose

Received by: ______________________________ Date: ________ (approval of completed log)

APPROVED
0120090141
DEC 18 2009
UMDNJ-NEWARK CAMPUS
INSTITUTIONAL REVIEW BOARD

UMDNJ
UNIVERSITY OF MEDICINE & DENTISTRY OF NEW JERSEY

SAINT BARNABAS
HEALTH CARE SYSTEM
A Legacy of Excellence

Do You Have Knee Pain from Osteoarthritis?

You may be eligible to receive two months of massage therapy at no cost to you.

If you suffer from confirmed osteoarthritis of the knee, you may qualify for participation in a clinical research study exploring the effects of massage therapy. The study is being conducted by UMDNJ in conjunction with the Saint Barnabas Ambulatory Care Center in Livingston, NJ.

To qualify, you must:

- Be 35 years of age or older
- In general good health
- Have knee pain secondary to osteoarthritis

Individuals who qualify will receive study-related services free of charge. Participation will be required for a six month period.

For information or to see if you qualify for this study, please call:

(973) 972-8564

APPROVED
0120090141
DEC 18 2009
UMDNJ-NEWARK CAMPUS
INSTITUTIONAL REVIEW BOARD

Exploring Massage Benefits for Arthritis of the Knee (EMBARK)

NIH Funding Mechanism: 1R01AT004623-01

Principal Investigator: Adam Perlman, MD, MPH, FACP

Draft or Version Number: 2.1

30 September 2009

APPROVED
01200901 4[illegible]
DEC 18 2009
exp 6-7-10
UMDNJ-NEWARK CAMPUS
INSTITUTIONAL REVIEW BOARD

STATEMENT OF COMPLIANCE

The study will be carried out in accordance with Good Clinical Practice (GCP) as required by the following *(use applicable regulations depending on study location and sponsor requirements; samples follow)*:

- United States (US) Code of Federal Regulations (CFR) applicable to clinical studies (45 CFR Part 46; 21 CFR Part 50, 21 CFR Part 56, and 21 CFR Part 312)
- ICH E6; 62 Federal Register 25691 (1997)
- NIH Clinical Terms of Award

All key personnel at the University of Medicine & Dentistry of New Jersey (UMDNJ), Saint Barnabas Health Systems and Yale University/Griffin Hospital (all individuals responsible for the design and conduct of this study) have completed Human Subjects Protection Training.

SIGNATURE PAGE

The signature below constitutes the approval of this protocol and the attachments, and provides the necessary assurances that this trial will be conducted according to all stipulations of the protocol, including all statements regarding confidentiality, and according to local legal and regulatory requirements and applicable US federal regulations and ICH guidelines.

Site Investigator:*

Signed: ______________________________ Date: ______________

Name Adam Perlman, MD, MPH, FACP
Title: Chairperson, Department of Primary Care
Director, Institute for Complementary & Alternative Medicine (ICAM)

** The protocol should be signed by the local investigator who is responsible for the study implementation at his/her specific site; ie, if Investigational New Drug study, the individual who signs the Form FDA 1572.*

TABLE OF CONTENTS

Page
Statement of Compliance 2
Signature Page 3
Table of Contents 4
List of Abbreviations 6
Protocol Summary 7
1 Key Roles 9
2 Background Information and Scientific Rationale 11
2.1 Background Information 11
2.2 Rationale 17
2.3 Potential Risks and Benefits 18
2.3.1 Potential Risks 18
2.3.2 Known Potential Benefits 19
3 Objectives 20
3.1 Study Objectives 20
3.2 Study Outcome Measures 21
3.2.1 Primary Outcome Measures 21
3.2.2 Secondary Outcome Measures 23
4 Study Design 25
5 Study Enrollment and Withdrawal 27
5.1 Subject Inclusion Criteria 27
5.2 Subject Exclusion Criteria 28
5.3 Treatment Assignment Procedures 28
5.3.1 Randomization Procedures 30
5.3.2 Masking Procedures 30
5.3.3 Reasons for Withdrawal 30
5.3.4 Handling of Withdrawals 30
6 Study Intervention 31
6.1 Concomitant Medications/Treatments 32
7 Study Schedule 33
7.1 Screening 33
7.2 Enrollment/Baseline 34
7.3 Follow-up 35
7.4 Final Study Visit 35
7.5 Early Termination Visit 35
7.6 Allowable Visit Windows 36
8 Study Procedures/Evaluations 37
8.1 Evaluations 37
8.2 Laboratory Evaluations 38
8.2.1 Clinical Laboratory Evaluations 38

9 Assessment of Safety 39
9.1 Methods and Timing for Assessing, Recording, and Analyzing Safety Parameters 39
9.1.1 Adverse Events 39
9.1.2 Serious Adverse Events 40
9.2 Reporting Procedures 41
9.3 Type and Duration of Follow-up of Subjects after Adverse Events 41
9.4 Safety Oversight – Data and Safety Monitoring Plan 41
10 Clinical Monitoring 43
10.1 Site Monitoring Plan 43
11 Statistical Considerations 44
11.1 Study Hypotheses 44
11.2 Sample Size Considerations 45
11.3 Final Analysis Plan 45
12 Source Documents and Access to Source Data/Documents 49
13 Quality Control and Quality Assurance 50
14 Ethics/Protection of Human Subjects 52
14.1 Ethical Standard 52
14.2 Institutional Review Board 52
14.3 Informed Consent Process 52
14.4 Exclusion of Women, Minorities, and Children (Special Populations) 53
14.5 Subject Confidentiality 53
14.6 Future Use of Stored Specimens 54
15 Data Handling and Record Keeping 55
15.1 Data Management Responsibilities 55
15.2 Data Capture Methods 56
15.3 Types of Data 56
15.4 Timing/Reports 56
15.5 Study Records Retention 56
16 Publication Policy 58
17 Literature References 59
SupplementS/Appendices 68
Appendix A: Schedule of Events 68

LIST OF ABBREVIATIONS

AE	Adverse Event/Adverse Experience
BMI	Body Mass Index
CAM	Complementary and Alternative Medicine
CDC	U.S. Centers for Disease Control
CFR	Code of Federal Regulations
CRF	Case Report Form
DHHS	Department of Health and Human Services
DSP	Data and Safety Monitoring Plan
EMBARK	Exploring Massage Benefits for Arthritis of the Knee
FWA	Federal-wide Assurance
GCP	Good Clinical Practice
HIPAA	Health Insurance Portability and Accountability Act
ICF	Informed Consent Form
ICH	International Conference on Harmonisation
ICMJE	International Committee of Medical Journal Editors
IRB	Institutional Review Board
MOP	Manual of Procedures
N	Number (typically refers to subjects)
NCCAM	National Center for Complementary & Alternative Medicine
NIH	National Institutes of Health
NSAIDS	Nonsteroidal Anti-inflammatory drugs
OA	Osteoarthritis
OCRA	Office of Clinical Research Affairs, NCCAM, NIH, DHHS
OHRP	Office for Human Research Protections
PCP	Primary Care Provider
PHI	Protected Health Information
PI	Principal Investigator
PRC	Yale/Griffin Prevention Research Center
QA	Quality Assurance
QC	Quality Control
ROM	Range of Motion
SAE	Serious Adverse Event/Serious Adverse Experience
SHRP	School of Health Related Professions
SOP	Standard Operating Procedure
UMDNJ	University of Medicine and Dentistry of New Jersey
US	United States
VAS	Visual Analog Pain Scale
WOMAC	Western Ontario Multipurpose Arthritis Center Index

PROTOCOL SUMMARY

**Title:**	Exploring Massage Benefits for Arthritis of the Knee (EMBARK)
**Phase:**	II
**Population:**	125 adult (≥ 35 yrs) subjects with diagnosed osteoarthritis of the knee, who meet the American College of Rheumatology criteria and are experiencing moderate pain (40-90 on Visual Analog Pain Scale) will be recruited from two (2) centers: Siegler Center for Integrative Medicine at the Saint Barnabas Ambulatory Care Center, Livingston, NJ; and the Integrative Medicine Center at Griffin Hospital, Derby, CT.
**Number of Sites:**	2
**Study Duration:**	24 months
**Subject Participation Duration:**	Six (6) months
**Description of Agent or Intervention:**	A usual care group and Four (4) arms - Manualized Swedish Massage – 30 or 60 minutes in duration, once a week for eight (8) weeks, Manualized Swedish Massage – 30 or 60 minutes in duration, twice a week for four (4) weeks and once a week for four (4) weeks.
**Objectives:**	Primary: Identify the optimal practical dose and treatment regimen of an 8 week course of Swedish massage therapy for OA of the knee. A Delphi Approach will determine the optimal dose/regimen(s) of the massage treatment groups listed above for OA of the knee. In addition to statistical comparisons of all outcomes of treatment arms to wait list, pair-wise comparisons between massage arms will be made. The Delphi panel will consider the magnitude of the statistical differences as well as potential cost/convenience (length & number of visits).<br><br>Secondary: Define the safety profile of an eight week course of Swedish massage for OA of the knee and to assess the duration of therapeutic effects of 8 weeks of Swedish massage on OA of the knee. Comparison of tabulated safety events by grade across massage treatment arms and against the wait-list control

arm will help identify any unforeseen consequences of the massage regimens. Therapeutic effect will be measured by validated outcome measures (WOMAC Global, Visual Analog Scale pain, range of motion and time to walk 50 feet) in patients with OA of the knee at the conclusion of therapy, and 2 and 4 months post therapy (8, 16, and 24 weeks post baseline).

	Session Duration	
Session Frequency	30 Minutes	60 Minutes
Twice-weekly for 4 weeks, then once-weekly for 4 weeks	Cohort A	Cohort B
Once-weekly for 8 weeks	Cohort C	Cohort D

Estimated Time to Complete Enrollment: Ten (10) Months

Schematic of Study Design:

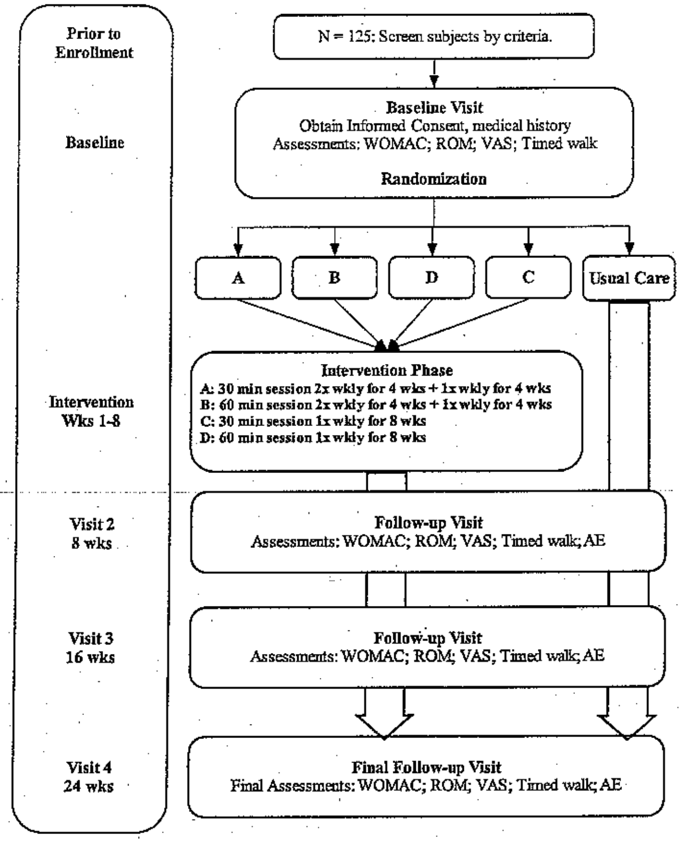

1 KEY ROLES

Refer to ICH E6, Section 6.1
(http://www.fda.gov/cder/guidance/959fnl.pdf).

Individuals:

Principal Investigator: Adam Perlman, M.D., M.P.H., F.A.C.P

Adam Perlman, M.D., M.P.H., F.A.C.P (PI)
65 Bergen St., Room 160
Newark, NJ 07101
(973) 972-8519
(973) 972-5572
a.perlman@umdnj.edu

Co-Investigator: David Hom, MS
65 Bergen St., Room 171
Newark, NJ 07101
(973) 972-8699
(973) 972-5572
homdl@umdnj.edu

Study Coordinator: Carl Milak
65 Bergen St., Room 160
Newark, NJ 07101
(973) 972-8564
(973) 972-5572
milakcj@umdnj.edu

Yale-Griffin Prevention Research Center
Principal Investigator: David Katz, M.D., M.P.H.
130 Division St.
Derby, CT. 06418
(203) 732-7194
(203_ 732-1264
David.katz@yale.edu; katzdl@pol.net

Co-Investigators:

Ather Ali, N.D., M.P.H.
130 Division St.
Derby, CT 06418
(203) 732-1265 x210
(203) 732-1264
Ather.ali@yale.edu

Valentine Nijke, M.D, M.P.H
130 Division St.
Derby, CT 06418
(203) 732-1265 x304
(203) 732-1264
valentine.njike@yalegriffinprc.org

Institutions:

UMDNJ-SHRP-ICAM
65 Bergen St., Room 160
Newark, NJ 07101

St. Barnabas Ambulatory Care Center – Siegler Center for Integrative Medicine
200 South Orange Ave.
Livingston, NJ 07039

Yale-Griffin Prevention Research Center
Griffin Hospital
130 Division St.
Derby, CT 06418

2 BACKGROUND INFORMATION AND SCIENTIFIC RATIONALE

2.1 Background Information

Toll of Osteoarthritis

Osteoarthritis (OA) is the most frequently reported chronic condition in the elderly, and by 2020, more than 50 million Americans will have OA[1, 3, 12]. The most common form of arthritis, OA becomes more prevalent with advancing age, but also commonly occurs in younger people with a history of joint injury[13-16]. Approximately 3 out of every 100 Americans below age 45, more than a quarter of Americans between the ages of 45 and 64, and 50-75% of Americans 65 and older, suffer from this debilitating disease[1, 16-18].

The costs of osteoarthritis in terms of human suffering are extremely high. Conventional therapies for OA have limited effectiveness and the toxicities associated with suitable drugs for long-term use often limit their utilization[4-6]. This leaves many patients with the undesirable options of surgery or facing chronic, often debilitating, daily pain and loss of function[5, 19-21]. The burden of OA, particularly associated with inadequate pain control, includes overall decreased quality of life, cognitive function, and sleep; interference with social relationships and activities of daily living; and increased depression and anxiety [22, 23]. Sleep disturbance due to pain is a common problem. In a study of adults with chronic pain due to OA of the knee, 31% experienced delayed sleep onset, 81% had problems with sleep maintenance, and 51% had at least weekly early morning awakenings due to pain[24]. Clearly, interventions that lead to better pain control would reduce suffering and improve quality of life for millions of Americans.

Osteoarthritis imposes a large economic burden on the U.S. through the morbidity and time loss it causes patients and the health resource utilization required in its management[25]. Estimates suggest that the cost of the care for osteoarthritis and its complications was $60 billion in 2004[25]. Even this is an underestimate, as cost studies for OA have focused only on the direct costs to the health-care system or on direct out-of-pocket expenditures by patients [12, 26-30]. Few studies have assessed the indirect patient costs of OA attributable to lost or foregone income and leisure time, or costs attributable to informal caregiver time [31, 32]. OA has major negative impacts on the American economy. OA pain is a leading cause of disability in the US, with annual cost of $7.11 billion in lost productive work time, 66% of which is due to chronic pain[33]. The burden on the social security system is also great, with OA the second leading cause for receiving disability benefits [3, 22].

Pathogenesis and Disease Mechanisms

<u>OA Disease:</u> OA is a slowly progressive degenerative disease of the joints that afflicts approximately 40 million Americans[1-3]. OA of the hip or knee is particularly disabling because it limits ambulation, but the affliction also strikes the hands, the spine, and the feet, with the same destructive joint process [5, 13, 21, 25]. Chronic pain, limitation of mobility affecting daily functioning, depression, muscle weakness and lack of stamina are all frequently experienced[20,

21, 25]. The endpoint of the OA disease process is total loss of joint cartilage in the affected area and the need for joint replacement[5, 25].

Subchondral bone thickening and increased cartilage turnover: Narrowing of the joint space, associated with destruction of articular cartilage and abnormal outgrowths of the subchondral bone, is one of the hallmarks of the disease and is used in radiological diagnosis[2, 34]. Human and animal studies indicate that increased subchondral bone and cartilage turnover are early events[5, 34, 35]. Changes in the composition of articular cartilage proteoglycans (e.g. chondroitin sulphate isomers and novel epitopes) are seen prior to cartilage fibrillation[19, 36, 37]. Subchondral bone stiffening, which may be secondary to subchondral cyst formation and healing, also occurs[34]. This decreased plasticity of the underlying bone results in less ability of the joint to respond to chronic and acute changes in load, and to increased sheer stresses at the bone-cartilage interface[34]. Research in humans and the major OA animal models of rhesus and cynomolgus macaque (monkeys) and the Duncan Hartley strain of guinea pigs, indicate that subchondral bone thickening precedes the cartilage damage[34, 35].

Ligament damage: There are changes in the ligaments associated with OA, and research points to ligament damage at the insertion point on subchondral bone as a possible initiating event for OA[34, 35]. Indeed, there is a high correlation of anterior cruciate ligament damage with OA of the knee, and OA in younger people is associated with a history of injury to the affected joint [14, 15, 34]. Changes in the direction or degree of tension on the bone following severe or subclinical damage to ligaments may serve as a stimulus to bone remodeling[34, 35, 38].

Muscle function: The muscles surrounding the joint also play a role in early and later stages of the disease. Compensatory stiffening of muscles occurs to protect the joint when it is injured[39]. However, this stiffening may persist after the initial inflammation has resolved, causing pain and uneven muscle tension across the joint[39]. The hamstrings and quadriceps stabilize the knee, distributing weight across the cartilage, and protecting the knee against injury[20]. Individuals with OA of the knee have been found to have less strength for flexion and extension, and decreased endurance and velocity of contraction, particularly at long muscle lengths, for the quadriceps and hamstrings, compared to age matched controls[20]. This has substantial negative impacts on activities of daily life such as walking, rising from a chair and climbing stairs[20, 21]. The inflammation and pain of OA can lead to reduced activity, further compromising neuromuscular function, joint stability and proprioception, and increasing the tendency for further injury[20, 39].

Inflammation: When there is tissue damage (for instance as may occur in a joint following mechanical stress or ligament damage), mononuclear phagocytic cells secrete inflammatory cytokines such as TNF-α and IL-1; and IL-6, nitric oxide, reactive oxygen species, and chemokines, are produced locally by a variety of cells[40-43]. Il-6 causes local and systemic effects, including hypothalamic pituitary axis (HPA) stimulation and increased circulating CRP[43]. Activation of the clotting and complement systems stimulates platelets and mast cells to release pro-inflammatory histamine and 5-HT (serotonin), and generates bradykinin from the kinin system[40]. Vascular permeability and migration of leukocytes is further increased, and mast cells release the slow reacting inflammatory mediators, leukotrienes and prostaglandins[40, 44]. Prostaglandin E2 (PGE2) is produced via breakdown of arachadonic acid by the cyclo-oxygenase pathway 2 (COX-2)[40]. It is this enzyme and pathway that is the target of action of COX-2 inhibitors and nonsteroidal anti-inflammatory drugs (NSAIDS), used to treat inflammation associated pain [45, 46]. PGE2 causes vasodilation, and potentiates the increased vascular permeability produced by histamine and bradykinin[44]. Activated monocytes produce chemokines and leukotrienes,

particularly LTB4 from arachadonic acid which attracts neutrophils and synergizes with PGE2 in increasing vascular permeability. Chronic inflammation with mononuclear and T cell infiltration, can develop if damage continues, or through presentation of self antigens in autoimmunity, or with persistent infection[46].

Current Conventional Treatments and their Limitations

Conventional treatments for OA include pain medications, exercises, hot/cold therapy, steroid injections and eventually, surgery to repair the joint [5, 25]. Despite conventional treatment, OA is a progressive disease that frequently leads to chronic pain and disability. In addition to their expense, pharmacologic agents are limited by toxicities to the gastrointestinal tract, liver and other organs[4-6]. In a survey of 3,100 OA patients, 25% were unsatisfied with their pain medications, 17% mainly due to side effects, and 63% primarily because of incomplete pain coverage. Drugs utilized for OA chronic pain management include acetaminophen (APAP), non-selective non-steroidal anti-inflammatory drugs (NSAIDS), COX-2 inhibitors, and opiod analgesics[45, 46].

Acetaminophen: N-acetyl-p-aminophenol (APAP, Tylenol), often a first-line analgesic for mild to moderate OA pain, inhibits prostaglandin synthesis and pyretic activity by an incompletely understood mechanism[80]. Hepatic and renal toxicites may occur if the 4000mg/day dosage is exceeded [45, 46, 80]. Because APAP is present in many other medications, patients may accidentally exceed this upper limit[45]. Another limitation is its lack of effectiveness in many OA patients[80]. In a Cochrane Review of the use of APAP for OA, only 5 of 7 RCTs showed superiority of APAP over placebo, and the treatment effect for pain was statistically significant, but small[80]. The need for multiple timed dosing, which may lead to incomplete pain coverage due to late or missed doses is another drawback to APAP [45].

NSAIDS: Non-steroidal anti-inflammatory drugs (NSAIDS) are generally used when APAP is inadequate to cover pain, and have been shown to be superior to APAP in comparative RCTs and patient surveys [45, 80]. The non-selective NSAIDS, such as aspirin, ibuprofen and naproxen, inhibit both COX-1 and COX-2 pathways which produce prostaglandins [45, 46, 81]. COX-1 is expressed constitutively in nearly all tissues and is involved in mediating normal platelet function, protecting gastric mucosa, and regulating renal blood flow [45, 81]. COX-2 is strongly upregulated in inflammation and mediates inflammation, pain and fever [45, 81]. The major toxicity associated with non-selective NSAIDS are gastrointestinal in nature[45]. Annual hospital admissions (approx. 107,000) and deaths (16,500) of arthritis patients in the US due to NSAID gastrointestinal events, were high before the introduction of selective COX-2 inhibitors[45, 46]. While these numbers have dramatically decreased, thousands still suffer with these side effects[45, 46]. Decline in renal function and increased risk of CVD events, including myocardial infarction, and congestive heart failure (10.5 fold increased risk in elderly patients with history of heart disease), are other serious side effects which increase with time on therapy and are associated with non-aspirin NSAIDS and COX-2 inhibitors [45, 46].

COX-2 inhibitors: COX-2 inhibitors have more favorable gastrointestinal side effect profiles than non-selective NSAIDS, but their labeling still warns of increased risk of GI toxicity[45, 46]. COX-2 inhibitors have been associated with increased risks of stroke and myocardial infarction, and Rofecoxib and Valdecoxib were withdrawn from the market because of these concerns [45, 46]. These side effects and risks of hypertension, acute renal failure or impaired renal function, make their use by persons at risk for, or suffering from, congestive heart failure, MI, stroke, or renal

failure, not recommended, or at least problematic[45, 46]. Unfortunately, and importantly, there is significant overlap in the populations who most need pain therapy for OA and those who are at increased risk of side effects from standard pharmacologic therapy. In addition, the decrease in prescriptions and use of NSAIDS and COX-2 inhibitors creates a gap in pharmacologic choices, and may be contributing to the under-treatment of chronic pain, and OA pain in particular[45, 46].

Opiod analgesics: In patients with side effects or risk factors that preclude continued usage of APAP, NSAIDS, and COX- 2 inhibitors, or when OA pain has progressed to a point where breakthrough pain is severely limiting quality of life, opiod analgesics may be used [45, 46]. Recent reviews of the use of opiods for chronic pain, and specifically OA pain treatment, are available[45, 46]. Briefly, these drugs block pain signals by binding to the mu, kappa, or delta opiod receptor in the central nervous system and fall into agonist, partial agonist, agonist-antagonist, and antagonist classes[45, 46]. Short half life opiods are often used for moderate pain in low doses with aspirin, APAP or ibuprofen. Short to medium half-life opiods may be used for severe pain relief. Limitations/concerns/side effects include; tolerance and need to increase dose, respiratory depression, cognitive impairment, myoclonus, constipation, nausea, emesis, somnolence, sexual dysfunction, and urinary retention[45, 46]. Renal dysfunction is a contraindication for many opiods, due to subsequent drug accumulation[45, 46]. For scheduled opiods, concerns and potential toxicity include dependence, addiction, and diversion of drugs to others; leading to under prescribing and sub-optimal dosing [45, 46].

Summary: Incomplete pain coverage, side effects, and recent well publicized events such as the multiple law suits and removal of COX II inhibitors from the market, have lessened the public's confidence in conventional options and has lead to increased interest in therapeutic interventions with fewer known and potential toxicities[82, 83]. Massage therapy, and certain other CAM interventions, are being utilized by OA sufferers and represent attractive, potentially effective therapies.

Summary of Pilot Trial

The University of Medicine & Dentistry of New Jersey, School of Health Related Professions (UMDNJ-SHRP) in collaboration with the Yale-Griffin Prevention Research Center (PRC) completed a randomized trial evaluating massage therapy for osteoarthritis of the knee [8]. This pilot trial, supported by CDC grant SIP-14-00, enrolled 68 subjects randomized to either massage intervention or wait-list. This pilot study demonstrated the efficacy of massage therapy in the treatment of OA of the knee. It is very important to note that the benefits of decreased pain and improved function persisted at the follow up time point, 8 weeks after the end of the massage treatment intervention. This supports the hypothesis that massage is well tolerated, efficacious and has the potential for long term benefits in patients with OA of the knee. Results of this trial were presented at the North American Integrative Medicine Research Conference in 2006 and published in the Archives of Internal Medicine that same year [8]. This successful pilot study lends clear and compelling support to a definitive RCT designed to determine optimal dosage of massage treatment, and further investigate sustainability of effect, therapeutic efficacy, and mechanism of action of this potential adjunctive therapy for OA of the knee.

PRELIMINARY STUDIES

Pilot Study: CDC SIP-14-00 and Prevention Research Center (U48-CCU115802)
In 2004, the University of Medicine & Dentistry, School of Health Related Professions (UMDNJ-SHRP) in collaboration with the Yale-Griffin Prevention Research Center (PRC) completed a randomized trial evaluating massage therapy for osteoarthritis of the knee [8].

The goal was to evaluate the safety and efficacy of a one-hour Swedish massage given twice weekly in weeks 1-4 and once weekly in weeks 5-8, as compared to control (delayed intervention), among adults with radiographically confirmed OA of the knee. Efficacy was based on a clinically significant (20 point) improvement in the Western Ontario and McMaster Universities Osteoarthritis index (WOMAC) pain and functional scores, and similar improvement in the visual analog scale (VAS) of pain assessment at the completion of the massage course of treatment (wk 8) and two months post-intervention (wk 16). Adults with radiographically established OA of the knee who met American College of Rheumatology criteria[120] and had a pre-randomization score of 40 to 90 on the Visual Analog Pain Scale (0 – 100 mm scale) were eligible. Sample size was set at 66 subjects total to achieve a statistical power of 80% (α=.05) to detect a 20 point difference between the two treatment arms at 8 weeks in the change on the WOMAC and VAS measures for walking pain.

Two licensed massage therapists used a standard Swedish full-body (not including the head and with extra attention to the knee area) therapeutic massage technique following a standard protocol for study intervention. The initial treatments (weeks 1-4) were given twice-weekly, followed by once-weekly sessions for weeks 5-8. The control group continued to receive standard medical care during weeks 1-8 and then crossed over to receive massage during weeks 9-16.

Two hundred ten (210) volunteers were screened for eligibility and enrollment at the Siegler Center for Integrative Medicine, St. Barnabas Medical Center, of whom 68 met eligibility criteria and were consented and randomized for participation. Approximately 82 (39%) were screened ineligible and 60 (28%) were unable to complete screening or were uninterested in further participation. Baseline demographic and clinical characteristics were comparable across the two groups - mean age of 70(11) years; body mass index of 28(8), 79% female, and 85% white. Extent of disability was comparable among all measured scales (WOMAC stiffness, 52.3(21.4); WOMAC functionality, 49.2(21.6); WOMAC global, 47.6(19.9), VAS pain score, 63(18); range of motion, 116(13) degrees; and time to walk 50 ft, 16(8) seconds. Only WOMAC pain score was slightly higher in the intervention arm [52.10(18.82)] versus the control arm [40.69(20.01)] at baseline (p=.02).

Table 1: Change in Outcome Measures from Baseline at 8 and 16 Weeks								
	At 8-wks				At 16-wks		Pooled Analysis	
	Intervention (n = 34)	p	Control (n = 34)	p	Control (n = 34)	p	Both (n = 68)	P
WOMAC score, mm								
Pain	-23.19 (24.30)	<.001	-3.08 (17.58)	.320	-13.52 (21.49)	<.001	-18.36 (23.28)	<.001
Stiffness	-21.60 (26.99)	<.001	-4.29 (24.18)	.310	-11.66 (30.13)	.03	-16.63 (28.82)	<.001
Functionality	-20.50 (22.50)	<.001	-5.02 (16.37)	.080	-14.04 (26.02)	.003	-17.27 (24.36)	<.001
Global	-21.15 (22.46)	<.001	-4.56 (15.85)	.100	-13.73 (24.48)	.002	-17.44 (23.61)	<.001
VAS pain score, mm	-22.59 (25.97)	<.001	-1.97 (21.07)	.590	-16.18 (30.24)	.004	-19.38 (28.16)	<.001
Range of motion, d	7.15 (11.45)	.001	-1.06 (14.20)	.670	-0.01 (14.78)	.990	3.57 (13.61)	.030
Time to walk 50 ft, s	-1.77 (2.73)	<.001	0.24 (4.81)	.770	0.03 (7.09)	.980	-0.87 (5.41)	.190

The mean (SD) WOMAC global score improved significantly (55%) from baseline value (p<.001), as did the score in each domain (pain, stiffness, and physical functional disability) (see Table 1). The greatest improvement from baseline in the intervention group was observed in pain, followed by stiffness and physical function (p<.001), while no significant change was observed in the control group from baseline in any of the domains. A similar response to treatment was observed in the VAS and the clinical assessment for range of motion. Findings persisted after controlling for demographic and baseline clinical values. The control group received the massage intervention delayed by 8 weeks and thus became a second intervention group during weeks 9 to 16. Pooled analyses of the two groups at 8 weeks post intervention demonstrate consistent improvements across all parameters, except time to walk 50 feet.

Table 2: Comparison between Improvements Observed in the Intervention and Control Groups			
	Control at 8-wk Follow-up (n = 34)	Intervention At 16-wk Follow-up (n = 34)	p
WOMAC score, mm			
Pain	-3.08 (17.58)	-18.52 (22.51)	.002
Stiffness	-4.29 (24.18)	-15.51 (22.28)	.050
Functionality	-5.02 (16.37)	-17.05 (20.15)	.009
Global	-4.56 (15.85)	-17.23 (19.88)	.005
VAS pain score, mm	-1.97 (21.07)	-17.15 (21.27)	.004
Range of motion, deg	-1.06 (14.20)	3.88 (13.61)	.150
Time to walk 50 ft, s	0.24 (4.81)	-2.28 (3.96)	.020

Results in Table 2 demonstrate the sustained and significant improvement in WOMAC domains by the intervention group at 8 week post intervention, as compared to the control group, who received conventional medical care for eight weeks. While demonstrating a significant clinical and statistical improvement in WOMAC domains, the massage therapy intervention was extremely safe, with only one report of increased discomfort, leading to discontinuation of therapy.

Summary:

This pilot study suggested the efficacy of massage therapy in the treatment of OA of the knee, with benefits persisting for eight weeks following treatment cessation. Massage was well tolerated,

decreased pain and improved function. The study was limited in that the subject pool was homogeneous; the intervention was delivered at one clinical site; only one 'reasonable' dosing regimen of massage was tested; and follow-up was limited to 8 weeks. Further study is needed to determine optimal dosage of massage treatment, sustainability of effect, confirm therapeutic efficacy, and establish the cost-effectiveness of this potential adjunctive therapy.

2.2 Rationale

Public Utilization and acceptance: There is widespread usage of CAM therapies by the public, despite the low level of insurance reimbursement for such interventions [84-87]. This is particularly prevalent for chronic diseases, such as OA, where conventional treatments fail to address underlying causes, fully relieve symptoms, or are associated with toxicity and other side effects [87]. According to a national survey conducted in 1997-1998, 30% of Americans greater than 65 years of age reported using alternative medicine (approximately 10 million, based on census data) and 19% (63 million visits, based on census data) visited an alternative medicine provider within the past year [88]. A variety of CAM therapies are being utilized by the public for rheumatic diseases and musculoskeletal disorders [8, 82, 89-93]. Some clinical trials have been performed to investigate efficacy [8, 91-94]. A review by Astin published in 2004 found evidence for efficacy of mind body therapies as adjunctive treatments in the management of RA and OA[90].The historical use of massage is documented in writings from many cultures, such as Ancient Greece, Rome, Japan, China, Egypt, and the Indian subcontinent [95]. The techniques described in this proposal are an outgrowth of the "Swedish Movement System" developed in the late 1700s by Per Henrik Ling, a Swedish fencer and gymnastics instructor [96]. Massage has become increasingly popular. According to a 2002 US survey, 5% of the 31,000 participants had used massage therapy in the preceding 12 months, and 9.3% had ever used it. Massage is generally used to relieve pain from musculoskeletal and other conditions, rehabilitate sports injuries, reduce stress, increase relaxation, decrease feelings of anxiety and depression, and aid general wellness[82, 95].

Availability and Safety: Swedish massage was chosen for the initial pilot in this trial due to its perceived effectiveness as well as general availability. Swedish massage is taught at most massage schools, practiced by the majority of massage therapists, and is therefore readily accessible to much of the public. Massage therapy has a high safety, low adverse event profile when administered by trained massage therapists[83]. It is acceptable to patients, reduces stress, anxiety, and pain[39, 83].

Potential mechanisms: Massage may work through a variety of mechanisms. Increased blood circulation to the local area has been thought to be one of the outcomes and benefits of massage, and some studies support this effect [97]. Other outcomes and mechanisms of massage therapy's effectiveness which have theoretical and some clinical trial and pre-clinical/basic research support include localized or peripheral effects such as decreasing muscle strain, balancing muscle tension across the joint, increased blood flow, lymphatic circulation, joint flexibility, proprioception, and positive mechanical changes in muscles [95, 97, 98]. Muscular force transmission is affected by fascial connections between neighboring muscles and between muscle and fascia spanning joints distal to the muscular insertion; this may provide additional mechanical basis for the effects of whole body massage on persons with localized knee OA [99, 100]. In addition, central effects such as stress reduction/relaxation, shift toward more parasympathetic nervous system activation, increased secretion of endorphins and serotonin, decreased substance P, improved sleep, blocking pain signals, and positive immune system effects, have also been demonstrated [95, 97, 98, 101-103].

Massage therapy affects on pain: Pain is one of the most difficult and debilitating symptoms of osteoarthritis. Massage therapy has been evaluated and found to have effectiveness as an adjunct treatment for pain secondary to cancer[104, 105], low back pain syndrome[106-108], rheumatoid arthritis[109], and fibromyalgia[102, 110, 111]. It also has been shown to be beneficial for patients with chronic pain following spinal cord injury[97]. In a randomized, open-label clinical trial, a series of classical Swedish massage therapy sessions was found to be as effective as conventional analgesic therapy for chronic rheumatic pain[82]. A recent study of massage for OA of the hand and wrist by Field et al. showed significant decreases in pain, depression and anxiety, with increased grip strength, compared to baseline and standard care control group [112]. Massage was performed once weekly for 4 weeks by a therapist, with instruction and encouragement to perform self treatment daily. Assessments were done before and after treatment at baseline and at 4 weeks[112]. The observation in our pilot trial of massage for OA of the knee that decreased pain and increased function persisted at least eight weeks after treatment cessation indicates lasting changes secondary to the use of massage for this disease indication. The design of this trial with 16 and 24 weeks post baseline follow ups, including biomarkers, will further characterize the durability and nature of these positive changes and contribute to the understanding of this important CAM intervention.

Massage therapy offers significant clinical benefits, including the potential to alleviate OA symptoms in all ages, providing benefits in terms of comfort, quality of life, functionality, safety, costs of care, and productivity. These benefits have not, to date, been adequately assessed for OA, although they are strongly supported by our pilot data (See B.6 below). Potential mechanisms of action of massage including stress reduction, positive modulation of the immune system, increased blood and lymphatic circulation, and improved joint function, usage, and proprioception; leading to reduced pain, strain and further injury, are consistent with the goals of improving symptoms, modulating disease status, and ultimately affecting disease course for OA.

Proposed is a dual-site, randomized, dose-ranging trial to determine the role and practice parameters for massage in the standard clinical management of OA based on investigation of efficacy and mechanism of action. The study hypothesis is that an 8 week course of Swedish massage therapy will be effective in reducing pain and improving function in patients with confirmed OA of the knee. A future phase III trial is planned to test the optimal massage intervention identified in this study against a validated sham/control intervention, incorporating all pertinent outcome measures from the antecedent studies. In addition, this study will further the development of robust methodologies to test the efficacy of massage interventions in general. The research agenda advanced by the current study will culminate with the establishment of the proper place for massage therapy among standard treatment options for the millions of Americans suffering with osteoarthritis.

2.3 Potential Risks and Benefits

2.3.1 Potential Risks

Potential for physical risk related to massage therapy are minimal, however each participant will be assessed by the massage therapist immediately following the session and by the study coordinator and investigator during follow-up for any adverse effects due to massage intervention.

All patient information used for research purposes will be strictly maintained according to HIPAA guidelines to ensure patient confidentiality at all times. Access to patient information will be limited to appropriate study personnel and all data will be coded to maintain confidentiality. Participants will be evaluated post-treatment to assess and monitor their condition. All documents with patient names will be kept in locked file cabinets and encrypted Microsoft Excel worksheets in password-protected computers.

At intake, all study participants will be asked to identify a physician with whom clinical findings of concern can and should be shared. Pertinent HIPAA requirements will all be addressed. The elucidation of any condition warranting clinical attention, whether by virtue of questionnaire or blood test, will result in notification of the subject's PCP. In uncertain cases, or when a PCP has not been identified, the case will be referred to the IRB.

2.3.2 Known Potential Benefits

Participation in this study may result in an intervention that improves the subjects' osteoarthritis and/or quality of life, and may thus benefit the research subject personally. However, it is possible that this study may be of no direct benefit to the study subject. The information gathered by this study may add to the knowledge of how to effectively treat patients with osteoarthritis and therefore produce benefits in the future for people with osteoarthritis.

3 OBJECTIVES

3.1 Study Objectives

Proposed is a dual-site, randomized, dose-ranging trial to compare four dose/regimens in order to identify the optimal protocol for clinical practice. The study hypothesis is that an eight (8) week course of Swedish massage therapy will be effective in reducing pain and improving function in patients with confirmed OA of the knee. Subsequent studies in this line of inquiry will develop and validate a sham control for massage, test the optimal dose/regimen identified in this trial against that validated control; investigate mechanism of action and role of massage in the prevention of disease progression; compare alternative massage techniques; assess the utility of massage at other joints than the knee; and assess cost-effectiveness. The ultimate goal of this line of inquiry is to determine the appropriate role and practice parameters for massage in the standard clinical management of osteoarthritis.

Specific Aim #1:<u>To identify the optimal practical dose and treatment regimen of an 8 week course of Swedish massage therapy for OA of the knee.</u>

It is crucial to identify optimal dose and treatment parameters for an intervention before proceeding to large efficacy trials. This goal will be accomplished by comparing the efficacy of the following four regimens to usual care control: 1. Thirty minutes, once a week, 2. Sixty minutes, once a week, 3. Thirty minutes, twice a week for four weeks and once a week for four weeks, and 4. Sixty minutes, twice a week for four weeks and once a week for four weeks. The regimens were chosen as practical regimens that are commonly used in massage therapy and contain a range of doses, with one higher than the pilot study and two lower doses. In addition to comparison to usual care, these regimens will be compared to each other both quantitatively and qualitatively. These regimens were designed to investigate the variables of length of individual treatment, (thirty vs. sixty minutes at once and twice a week), frequency (one vs. two times/wk at thirty and sixty minutes), and total treatment time; 240 minutes (thirty once/wk x eight wks), 480 minutes (sixty once x eight wks), or 360 minutes (thirty twice/wk x four wks and once wk x four weeks) and 720 minutes (sixty twice/wk x four wks, and sixty once/wk for four wks.

A modified Delphi Approach will determine the optimal dose/regimen(s) of the massage treatment groups listed above for OA of the knee. In addition to statistical comparisons of all outcomes of treatment arms to usual care, pair-wise comparisons between massage arms will be made. The Delphi panel (pain specialist/ rheumatologist; integrative medicine physician: licensed massage therapist) will consider the magnitude of the statistical differences as well as potential cost and convenience (length and number of visits). For example, a small difference in efficacy would not justify a four-fold increase in potential cost (e.g. 30 minutes once per week vs. 60 minutes twice/week).

We hypothesize that this combination of assessments will allow the identification of an 'optimal' dosing regimen that confers meaningful functional benefits and symptom relief, is practical for routine application into clinical practice for treatment of OA of the knee, and serves as the basis for on-going research within this line of inquiry.

Specific Aim #2: **To define the safety profile of an eight week course of Swedish massage for OA of the knee.**

Safety and tolerability will be assessed through participant self-report of symptoms or changes in symptoms since baseline (obtained from daily adverse event logs and limited history and physical) after eight weeks of massage therapy among subjects with OA of the knee. Comparison of tabulated safety events by grade (mild, moderate, severe, life-threatening) across massage treatment arms and against the usual care arm will help identify any unforeseen consequences of the massage regimens.

We hypothesize that all of the massage therapy dose/regimens will be safe and well tolerated, based on our pilot trial data and the safety and acceptability record of therapeutic massage, as reported in the literature.

Specific Aim #3: **To assess the duration of therapeutic effects of 8 weeks of Swedish massage on OA of the knee.**

Therapeutic effect will be measured by validated outcome measures (WOMAC Global, Visual Analog Scale pain, range of motion and time to walk 50 feet) in patients with OA of the knee at the conclusion of therapy, and 2 and 4 months post therapy (8, 16, and 24 weeks post baseline).

We hypothesize, based on our pilot trial, that decreased pain and increased function will be present at the end of treatment and persist at least two months after cessation of massage treatment. The later time point of 4 months post therapy has been added to further assess durability of therapeutic effect and is exploratory.

Summary: The ultimate goal for the line of inquiry initiated by the current study is to determine the role and practice parameters for massage in the standard clinical management of OA based on investigation of efficacy and mechanism of action. A future phase III trial is planned to test the optimal massage intervention identified in this study against a validated sham/control intervention, incorporating all pertinent outcome measures from the antecedent studies. In addition, this study will further the development of robust methodologies to test the efficacy of massage interventions in general. The research agenda advanced by the current study will culminate with the establishment of the proper place for massage therapy among standard treatment options for the millions of Americans suffering with osteoarthritis.

3.2 Study Outcome Measures

3.2.1 Primary Outcome Measures

Western Ontario and McMaster Universities Osteoarthritis of the Knee and Hip Index (WOMAC). The WOMAC has been used extensively in the quantitative assessment of osteoarthritis of the knee, and proven to be effective in assessing pain in those suffering from the illness [9-11]. The Index is self-administered and assesses the three dimensions of pain, disability and joint stiffness in knee and hip osteoarthritis through 24 questions. WOMAC™ 3.1 is available in 65 languages using either a five-point Likert scale or a 100 mm visual analog scale. The WOMAC has

been subject to numerous validation studies to assess reliability and responsiveness to change in therapy, including physical forms of therapy[9-11].

After familiarizing the participant to the format of the questions and the 100 mm scale, the study coordinator trained in the protocol will assess responses to the WOMAC domains.

WOMAC Osteoarthritis Index

PAIN: Think about the pain you felt during the last 48 hours caused by arthritis in your knee.

**Question: How much pain have you had ...**					
	**None**	**Mild**	**Moderate**	**Severe**	**Extreme**
When walking on a flat surface?					
When going up or down stairs?					
At night while in bed? (that is – pain that disturbs your sleep)					
While sitting or lying down?					
While standing?					

STIFFNESS: Think about the stiffness (not pain) you felt during the last 48 hours caused by arthritis in your knee. Stiffness is a sensation of decreased ease in moving your joint.

	**None**	**Mild**	**Moderate**	**Severe**	**Extreme**
How severe has your stiffness been after you first woke up in the morning?					
How severe has your stiffness been after sitting or lying down or while resting later in the day?					

DIFFICULTY PERFORMING DAILY ACTIVITIES: Think about the difficulty you had in doing the following daily physical activities during the last 48 hours caused by arthritis in your knee. By this we mean your ability to move around and take care of yourself.

**Question: How much difficulty have you had ...**					
	**None**	**Mild**	**Moderate**	**Severe**	**Extreme**
When going down the stairs?					
When going up the stairs?					
When getting up from a sitting position?					

While standing?					
When bending to the floor?					
When walking on a flat surface?					

DIFFICULTY PERFORMING DAILY ACTIVITIES: Think about the difficulty you had in doing the following daily physical activities during the last 48 hours caused by arthritis in your knee. By this we mean your ability to move around and take care of yourself.

**Question: How much difficulty have you had ...**					
	**None**	**Mild**	**Moderate**	**Severe**	**Extreme**
Getting in or out of a car, or getting on or off a bus?					
While going shopping?					
When putting on your socks or panty hose or stockings?					
When getting out of bed?					
When taking off your socks or panty hose or stockings?					
While lying in bed?					

DIFFICULTY PERFORMING DAILY ACTIVITIES: Think about the difficulty you had in doing the following daily physical activities during the last 48 hours caused by arthritis in your knee. By this we mean your ability to move around and take care of yourself.

**Question: How much difficulty have you had ...**					
	**None**	**Mild**	**Moderate**	**Severe**	**Extreme**
When getting in or out of the bathtub?					
While sitting?					
When getting on or off the toilet?					
While doing heavy household chores?					
While doing light household chores?					

3.2.2 Secondary Outcome Measures

Pain: The Visual Analog Scale (VAS) is a well-validated [138] mechanical scale used to measure pain sensation intensity evoked by nociceptive stimuli [139]. Subjects quickly and easily rate stimuli within the series by indicating level of pain on a 100 mm scale. Pain intensity is represented by the participant drawing a line on the scale indicative of pain experienced (0 = no pain, to 100 = worse pain imaginable). The VAS is anchored at the left by "no pain sensation" and at the right by "the most intense pain sensation imaginable."

No Pain	______________________	Worst Pain Imaginable
Directions	Ask the patient to indicate on the line where the pain is in relation to the two extremes. Qualification is only approximate; for example, a midpoint mark would indicate that the pain is approximately half of the worst possible pain.	

Joint Flexibility: Joint flexibility is defined as the range of motion (ROM) allowed at the knee. The knee's ROM is measured by the number of degrees from the starting position of a segment to its position at the end of its full range of the movement. This is measured using a double-armed goniometer. A stationary arm holding a protractor is placed parallel with a stationary body segment and a movable arm moves along a moveable body segment. The pin (axis of goniometer) is placed over the joint. See figure [140].

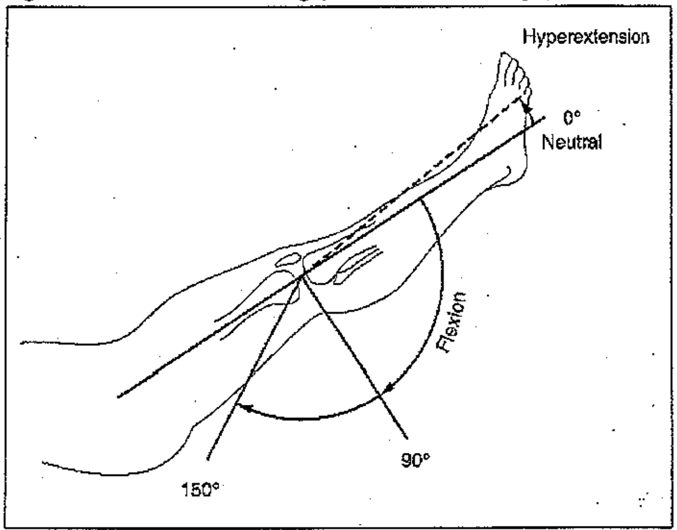

Physical function: Measured time in seconds to walk fifty (50) feet (15 m) on a level surface within the clinic facilities.

Limited Medical History: At screening and every follow-up visit participants will provide history of medication usage (prescription and over-the-counter), symptoms, duration of symptoms, date of diagnosis (screening), any change in health status and other relevant clinical information to the site study coordinator using a standardized data collection instrument.

Demographic Data: Standard demographic data (e.g. age, gender, height, weight) will be collected at baseline on all participants by the site study coordinator using a standardized data collection instrument.

Daily Concomitant Medication and Adverse Event Logs: All participants will be asked to collect and complete medication logs, indicating product used, dosage and duration of use. Study coordinators will provide calendar diaries and reminders to complete on daily any current use or change in medications and returned to the sites on a monthly basis. Questions about any experienced adverse events or changes in physical function/behavior will be collected to measure the safety of massage interventions. The study coordinator will remind participants on a regular basis through phone calls and mailings.

4 STUDY DESIGN

- ***Design:*** *Phase II dose-finding with short-term follow-up to determine efficacy and safety of Swedish Massage intervention on patients with confirmed OA of the knee.*
- ***Study Hypothesis:*** *That an eight (8) week course of Swedish massage therapy will be effective in reducing pain and improving function in patients with confirmed OA of the knee.*
- ***Participant Assignment:*** *Strafitied random assignment to one of four (4) massage arms and a usual care control group. The four (4) massage cohorts will be conducted for a period of eight (8) weeks and are: 1) thirty (30) minutes once/week, 2) sixty (60) minutes once/week, 3) thirty (30) minutes twice/week for four (4) weeks and once/week for four (4) weeks and 4) sixty (60) minutes twice/week for four (4) weeks and once/week for four (4) weeks.*
- ***Study population:*** *125 adult subjects (over 35 years of age) with radiographically diagnosed osteoarthritis of the knee, who meet the American College of Rheumatology criteria and are experiencing moderate pain (40-90 on the Visual Analog Pain Scale). They will be recruited from two (2) centers: Siegler Center for Integrative Medicine at the Saint Barnabas Ambulatory Care Center, Livingston, NJ, and the Integrative Medicine Center at Griffin Hospital, Derby, CT.*

Stratification: *Trial site (Siegler Center at Saint Barnabas Ambulatory Care Center or the Integrative Medicine Center at Griffin Hospital) and Body Mass Index (<> 25).*

Subject Participation: *Expected duration is six (6) months.*

- ***Follow-up Visits:*** *Upon completion of intervention, patients will be seen at 8, 16 and 24 weeks post randomization.*
- ***Study Endpoints:***

  Primary: *Improvement in Western Ontario and McMaster Universities Osteoarthritis of the Knee and Hip Index (WOMAC). The WOMAC has been used extensively in the quantitative assessment of osteoarthritis of the knee, and proven to be effective in assessing pain in those suffering from the illness [9-11]. The Index is self-administered and assesses the three dimensions of pain, disability and joint stiffness in knee and hip osteoarthritis through 24 questions.*

  Secondary: *2) the Visual Analog Scale (VAS), 3) Range of Motion (ROM)/Joint Flexibility, 4) Physical function (time to walk fifty feet), 5) Limited medical history, 6) Demographic data, and 7) Daily concomitant Medication and Adverse Event logs.*

- ***Analysis:*** An **intention-to-treat analysis** will be used to evaluate pre- and post-test scores to address the effects of the interventions on randomly assigned subjects whether or not

they complete the study. Using the principle of last observation carried forward, missing post-test scores will be filled with the test scores that will be collected closest to the time of dropout. Secondary analyses will include tests to determine whether the interventions are effective for those subjects who actually complete the study (i.e. per-protocol analysis).

In all analyses, a two-tailed α of less than 0.05 will be considered statistically significant. SAS software for Windows version 9.1 will be used to carry out all statistical analyses.

- **Expert Panel Review (Modified Delphi Technique):** At the completion of the trial, an independent three-member expert panel will be convened to review the results and determine the appropriate regimen to advocate for later phase III evaluation.

5 STUDY ENROLLMENT AND WITHDRAWAL

The total sample size will be a combined 125 volunteers between two sites: The Siegler Center for Integrative Medicine at the Saint Barnabas Ambulatory Care Center, Livingston, NJ, and The Inegrative Medicine Center at Griffin Hospital, Derby, CT..

Eligible female volunteers, as well as eligible minority volunteers will be approached for participation in this trial at both sites. Osteoarthritis of the knee is highly prevalent in women, as well as prevalent in all races and ethnic groups. Children will not be approached to participate in this trial as osteoarthritis of the knee is a chronic condition typically seen in the elderly population. In the earlier study, conducted solely at Saint Barnabas Medical Center, approximately 80% of the cohort was female and 14% non-white in ethnicity/race. Based on practice profile we anticipate a similar mix of participants (reflective of osteoarthritis epidemiology) at the Integrative Medicine Center at Griffin Hospital.

Study population will be drawn from the general public via IRB-approved information letters and advertisements, as well as from physicians through referrals. These advertisements, information letters and physician referrals will be focused on the target population (e.g., over age 35 with radiographically diagnosed osteoarthritis of the knee.

5.1 Subject Inclusion Criteria

Inclusion criteria (all inclusion criteria must be met):

1. 35 years of age or greater.
2. Written confirmation of OA of the knee as provided by the participant's physician.
3. Radiographically-established OA of the knee.
4. Pre-randomization score of 40 to 90 on the Visual Analog Pain Scale (0 – 100 mm scale).
5. Patients with bilateral knee involvement will have the more severely affected knee designated as the study knee.
6. American College of Rheumatology defined OA of the knee [120]; specifically:
    a. Knee pain
    b. Satisfaction of at least five of the following nine criteria:
        i. Age great than 50 years
        ii. Stiffness < 30 minutes
        iii. Crepitus
        iv. Bony Tenderness
        v. Bony enlargement
        vi. No palpable warmth
        vii. ESR < 40 mm/hr
        viii. Rheumatoid Factor (RF) < 1:40
        ix. Synovial Fluid signs of Osteoarthritis

5.2 Subject Exclusion Criteria

Exclusion criteria (no exclusion criterion may be present):

1. Presence of rheumatoid arthritis, fibromyalgia, recurrent or active pseudo gout.
2. Presence of cancer or other serious medical conditions.
3. Signs or history of kidney or liver failure.
4. Presence of asthma requiring the use of corticosteroid treatment.
5. Use of oral corticosteroids within the past four weeks.
6. Use of intra-articular knee depo-corticosteroids with the past three months.
7. Use of intra-articular hyaluronate with the past six months.
8. Arthroscopic surgery of the knee within the past year.
9. Significant injury to the knee within the past six months.
10. Presence of a rash or open wound over the knee.
11. Unable to satisfy the treatment and follow-up requirements.
12. Unable to provide written informed consent.
13. Currently receiving massage therapy on a regular basis (at least twice a month).
14. Knee replacement of study knee (ok if the knee not being studied has been replaced).

5.3 Treatment Assignment Procedures

Volunteers responding to IRB-approved information letters or advertisements will undergo a structured telephone interview administered by the study coordinator at each site to assess self-reported eligibility based on established inclusion criteria. A case report form will be used to record initial interview responses including demographics, brief medical history, and prescription medication/supplement use. This information will yield a result of eligible/ineligible/or uncertain potential study participant. In any uncertain cases, the candidate subject's status will be determined by the PI in consultation with the investigative team as required. Following prescreening questioning, a detailed description of the study will be presented to all eligible subjects. Subjects deemed eligible at the intake telephone interview, agreeing to the duration of the study and expected commitments, will be scheduled for an on-site evaluation, to provide informed consent and undergo clinical eligibility screening.

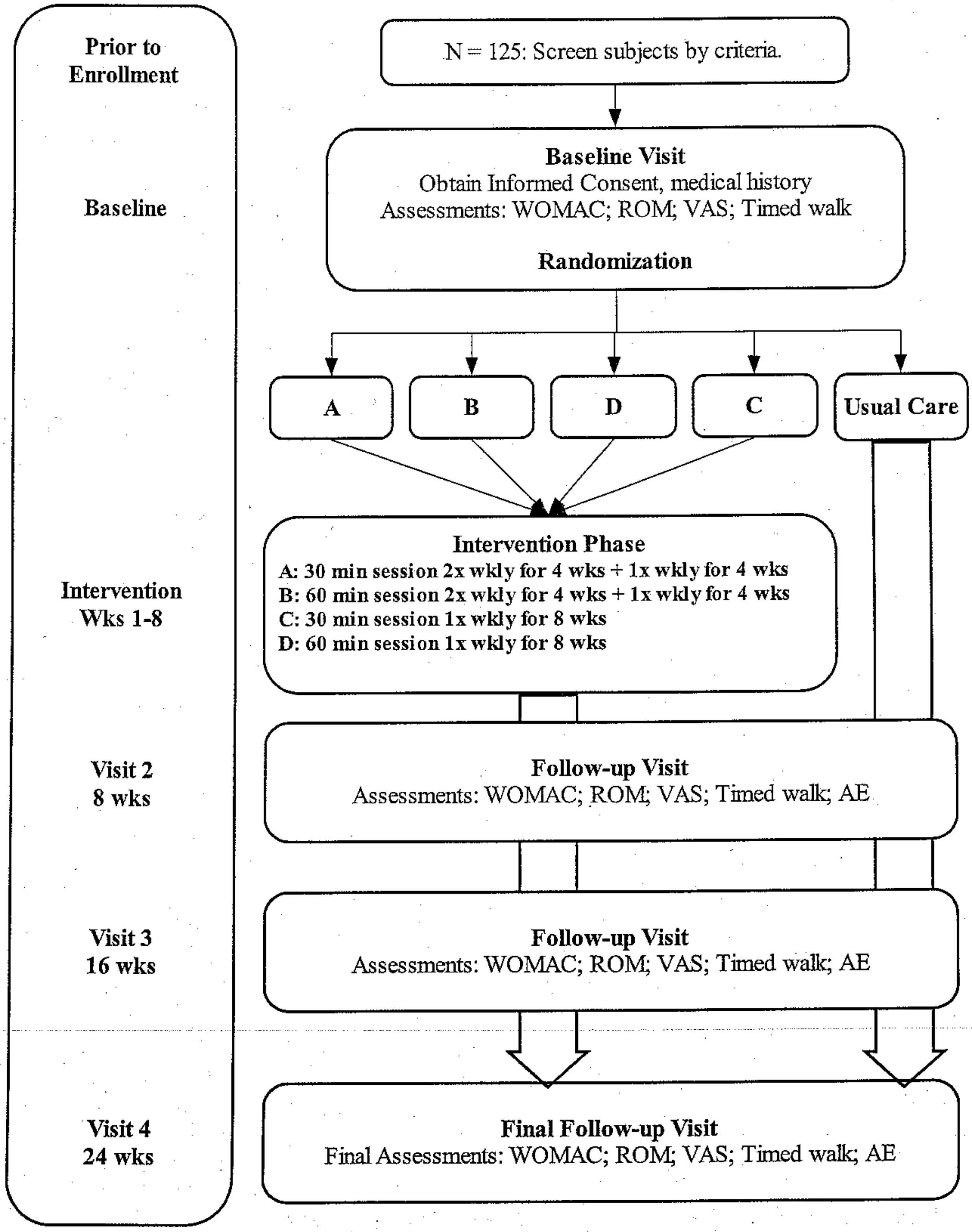
Prior to Enrollment
Baseline
Intervention Wks 1-8
Visit 2 8 wks
Visit 3 16 wks
Visit 4 24 wks
N = 125: Screen subjects by criteria.
Baseline Visit
Obtain Informed Consent, medical history
Assessments: WOMAC; ROM; VAS; Timed walk
Randomization
A
B
D
C
Usual Care
Intervention Phase
A: 30 min session 2x wkly for 4 wks + 1x wkly for 4 wks
B: 60 min session 2x wkly for 4 wks + 1x wkly for 4 wks
C: 30 min session 1x wkly for 8 wks
D: 60 min session 1x wkly for 8 wks
Follow-up Visit
Assessments: WOMAC; ROM; VAS; Timed walk; AE
Follow-up Visit
Assessments: WOMAC; ROM; VAS; Timed walk; AE
Final Follow-up Visit
Final Assessments: WOMAC; ROM; VAS; Timed walk; AE

5.3.1 Randomization Procedures

Upon satisfaction of screening criteria, the site coordinator will contact the participant to notify him/her of acceptance into the trial and arrange for the clinic coordinator to schedule a massage session based on the PRC data manager's randomization. The clinic coordinator will contact the Yale-Griffin PRC, who will allocate the subject to one of five arms and assign a study number using an SAS-generated random table. The study coordinator will be blinded to treatment assignment. Eligible participants for each site will be block randomized using a permuted block (blocks of 7 or 8) design in a 1:1:1:1:1 ratio and stratified by site, body mass index (BMI) to ensure balance between the intervention and usual care groups across the two performance sites. Usual care controls completing follow-up will be evaluated to confirm eligibility and offered gift certificates for six (6) 30 minute massages. Those assigned to the massage intervention will evaluated to confirm eligibility and will be offered gift certificates for two (2) 60 minute massages.

5.3.2 Masking Procedures

Participant assessments (e.g. Range of Motion, Timed walk of 50 feet) and interviews (e.g. Concomitant medications and Adverse Events) will be conducted by a study coordinator blinded to participant treatment assignment. Every effort will be made to maintain the treatment masking – study coordinator will not participate in appointment scheduling, assessments/ interviews done at a facility away from the locations where the massage sessions are conducted.

5.3.3 Reasons for Withdrawal

A study subject will be discontinued from participation in the study if:

- Any clinical adverse event (AE), intercurrent illness, or other medical condition or situation occurs such that continued participation in the study would not be in the best interest of the subject.
- Development of any exclusion criteria may be cause for discontinuation.

Subjects are free to withdraw from participating in the study at any time upon request.

5.3.4 Handling of Withdrawals

Study participants who withdraw from the study for any reason – adverse event, ineligibility, or voluntary withdrawal will be asked to complete one final study visit for completion of described assessment tools (WOMAC, VAS, Range of Motion, and Physical Function measures).

6 STUDY INTERVENTION

Massage Intervention: Licensed therapists certified by the National Certification Board for Therapeutic Massage and Bodywork will provide the massage therapy. The therapists will use a Swedish full-body (with special attention to knee area and not including head) therapeutic massage technique [129] and following a standard protocol for the study intervention (See Appendix 2). This includes skin rolling, cross-fiber friction, parallel friction, vibration and assisted breathing exercises in addition to:

- Petrissage - compression or manipulation of soft tissue between the fingers and thumb
- Effleurage - gliding of hands over the skin or soft tissues
- Tapotement - percussion-based massage where hands strike soft tissue in a repetitive, rhythmic fashion) techniques

Petrissage	Effleurage	Tapotement
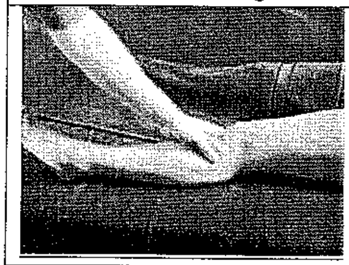	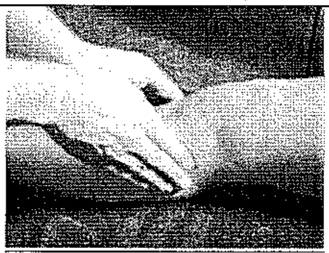	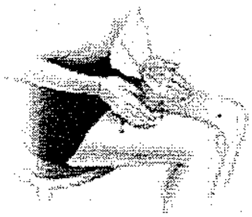

Massage sessions will be 30 minutes (cohorts 1 and 2) or 60 minutes (cohorts 3 and 4) in duration with participants in a supine or prone position during the session – flipping over mid-way through the session.

To minimize practitioner variability of massage, a detailed protocol incorporating specific strokes (petrissage, effleurage and tapotement) will be manualized by an expert panel prior to study initiation. Study staff will meet with the therapists at regular intervals to assure protocol compliance.

Following massage treatment, participants will be instructed to remain seated for approximately 10 minutes for observation of adverse reaction/side effects. Upon receiving clearance to leave by the massage therapist, participants will be given an appointment card for their next visit and instructed to call the site coordinator should they experience any unpleasant/unanticipated adverse effects between visits.

Massage Manualization Process: A panel of three experts in the field of massage therapy will be convened prior to study initiation to manualize the proposed massage intervention. Manualization was developed in the 1990's for the creation of treatment manuals for psychotherapy, both to help provide methodologic rigor in the evaluation of psychotherapy, and as a means to provide specificity and guidelines around individualized treatment[130, 131]. Massage as a therapy has similar characteristics as psychotherapy – need for methodologic rigor for clinical trials and to standardize patient-customized treatments and practitioner variation[132, 133]. Manualization was considered an integral methodologic component for rigorous research on CAM therapies by an IOM committee

in 2002 [134]. This panel will have broad clinical experience treating OA of the knee, be knowledgeable with massage therapy and familiar with implementing massage for treating OA of the knee for manual creation. This process will be overseen by Janet Kahn, PhD, Director of Research for the Massage Therapy Research Consortium, who will identify individuals from the Consortium to participate. The previously developed protocol will be used in the development of the manual. The panel will create a manual following the guidelines mentioned by Schnyer et al, as they originally related to acupuncture [135]. Assessment of Therapist Compliance with Study Intervention

The Treatment Manual will be distributed to the therapists for training and quality control purposes. Therapists will adhere to the protocol and will be periodically reviewed by the study teams for consistency. As part of the Manualization process a log sheet will be developed to address reproducibility and integrity of the protocol. The log will document the therapists' sessions and any deviation from the manual's steps.

6.1 Concomitant Medications/Treatments

Daily Concomitant Medication Logs: All participants will be asked to collect and complete medication logs, indicating product used, dosage and duration of use. Study coordinators will provide calendar diaries and reminders to complete on daily any current use or change in medications and returned to the sites on a monthly basis. The study coordinator will remind participants on a regular basis through phone calls and mailings.

7 STUDY SCHEDULE

7.1 Screening

Telephone Screening: Responding volunteers will undergo a structured telephone interview administered by the study coordinator at each site to assess self-reported eligibility based on established inclusion criteria. A case report form will be used to record initial interview responses including demographics, brief medical history, and prescription medication/ supplement use. This information will yield a result of eligible/ineligible/or uncertain potential study participant. In any uncertain cases, the candidate subject's status will be determined by the PI in consultation with the investigative team as required. Following prescreening questioning, a detailed description of the study will be presented to all eligible subjects. Subjects deemed eligible at the intake telephone interview, agreeing to the duration of the study and expected commitments, will be scheduled for an on-site evaluation, to provide informed consent and undergo clinical eligibility screening.

Informed Consent Process: A written informed consent form approved by each site's Institutional Review Board (IRB) will be used to obtain participant consent prior to clinical screening. Extensive discussion of risks and possible benefits of this therapy will be provided to the subjects and their families. Consent forms describing in detail the massage interventions, study procedures, and risks are given to the subject and written documentation of informed consent is required prior to starting intervention. Upon reviewing the document, the investigator will explain the research study to the subject and answer any questions that may arise. The subjects will sign the informed consent document prior to any procedures being done specifically for the study. Informed consent will be obtained by the study coordinator at each site. All participants will be informed of the option of not participating, and of stopping at anytime during the study. A copy of the informed consent document will be given to the subjects for their records.

The rights and welfare of the subjects will be protected by emphasizing to them that the quality of their medical care will not be adversely affected if they decline to participate in this study. Consent procedures will conform to HIPAA requirements and human subjects protection requirements of the participating institutional review boards.

Clinical Screening Evaluation: The clinical screening evaluation will consist of a set of structured questions following the eligibility criteria. Written confirmation of OA of the knee will be obtained by the participant's physician at the request of the site PI. Volunteers at each site found to be eligible in the clinical screening process and providing informed consent to participate in the study will be enrolled and undergo baseline evaluation, followed by randomization to treatment groups. Enrollment at both sites will continue until the requisite sample of 125 participants is reached.

List of Eligibility Criteria collected during Participant Screening:
Inclusion criteria (all inclusion criteria must be met):

1. 35 years of age or greater.
2. Written confirmation of OA of the knee as provided by the participant's physician.

3. Radiographically-established OA of the knee.
4. Pre-randomization score of 40 to 90 on the Visual Analog Pain Scale (0 – 100 mm scale).
5. Patients with bilateral knee involvement will have the more severely affected knee designated as the study knee.
6. American College of Rheumatology defined OA of the knee [120]; specifically:
    a. Knee pain
    b. Satisfaction of at least five of the following nine criteria:
        i. Age great than 50 years
        ii. Stiffness < 30 minutes
        iii. Crepitus
        iv. Bony Tenderness
        v. Bony enlargement
        vi. No palpable warmth
        vii. ESR < 40 mm/hr
        viii. Rheumatoid Factor (RF) < 1:40
        ix. Synovial Fluid signs of Osteoarthritis

Exclusion criteria (no exclusion criterion may be present):

1. Presence of rheumatoid arthritis, fibromyalgia, recurrent or active pseudo gout.
2. Presence of cancer or other serious medical conditions.
3. Signs or history of kidney or liver failure.
4. Presence of asthma requiring the use of corticosteroid treatment.
5. Use of oral corticosteroids within the past four weeks.
6. Use of intra-articular knee depo-corticosteroids with the past three months.
7. Use of intra-articular hyaluronate with the past six months.
8. Arthroscopic surgery of the knee within the past year.
9. Significant injury to the knee within the past six months.
10. Presence of a rash or open wound over the knee.
11. Unable to satisfy the treatment and follow-up requirements.
12. Unable to provide written informed consent.
13. Currently receiving massage therapy on a regular basis (at least twice a month).
14. Knee replacement of study knee (ok if the knee not being studied has been replaced).

7.2 Enrollment/Baseline

ENROLLMENT: Volunteers responding to IRB-approved information letters or advertisements will undergo a structured telephone interview administered by the study coordinator at each site to assess self-reported eligibility based on established inclusion criteria. A case report form will be used to record initial interview responses including demographics, brief medical history, and prescription medication/supplement use. This information will yield a result of eligible/ineligible/or uncertain potential study participant. In any uncertain cases, the candidate subject's status will be determined by the PI in consultation with the investigative team as required (See Section 7.1). Following prescreening questioning, a detailed description of the study will be presented to all eligible subjects. Subjects deemed eligible at the intake telephone interview, agreeing to the duration of the study and expected commitments, will be scheduled for an on-site evaluation, to provide informed consent and undergo clinical eligibility screening.

BASELINE: At baseline visit the subject assessments will include:

- WOMAC Index
- Visual Analog Pain Scale
- Joint Flexibility/Range of Motion
- Physical Function – time to walk fifty feet
- Limited Medical History

Upon satisfaction of eligibility criteria, study coordinator will collect informed consent from participant at this baseline visit. Randomization (see description in Section 5.3.1 – Randomization procedures) of consenting participants will be done per procedure and clinic schedulers will contact and schedule all future appointments with study participant to maintain blinding of study coordinator.

7.3 Follow-up

Follow-up visits occur at weeks 8 (separate from intervention session), 16 and 24.

Evaluations to be conducted at the each follow-up visit include:

- WOMAC Index
- Visual Analog Pain Scale
- Joint Flexibility/Range of Motion
- Physical Function – time to walk fifty feet
- Limited Medical History
- Collection of Daily Concomitant Medication and Adverse Event Logs

7.4 Final Study Visit

Final study visit is at week 24, four (4) months after the completion of last intervention session.

Evaluations to be conducted at the final visit include:

- WOMAC Index
- Visual Analog Pain Scale
- Joint Flexibility/Range of Motion
- Physical Function – time to walk fifty feet
- Limited Medical History
- Collection of Daily Concomitant Medication and Adverse Event Logs

7.5 Early Termination Visit

Study participants who withdraw from the study for any reason – adverse event, ineligibility, or voluntary withdrawal will be asked to complete one final study visit for completion of described assessment tools:

- WOMAC Index
- Visual Analog Pain Scale

- Joint Flexibility/Range of Motion
- Physical Function – time to walk fifty feet
- Limited Medical History
- Collection of Daily Concomitant Medication and Adverse Event Logs

7.6 Allowable Visit Windows

Allowable windows will be plus or minus 10 days around each scheduled clinic visit and each massage visit.

8 STUDY PROCEDURES/EVALUATIONS

8.1 Evaluations

Western Ontario and McMaster Universities Osteoarthritis of the Knee and Hip Index (WOMAC). The WOMAC has been used extensively in the quantitative assessment of osteoarthritis of the knee, and proven to be effective in assessing pain in those suffering from the illness [9-11]. The Index is self-administered and assesses the three dimensions of pain, disability and joint stiffness in knee and hip osteoarthritis through 24 questions. WOMAC™ 3.1 is available in 65 languages using either a five-point Likert scale or a 100 mm visual analog scale. The WOMAC has been subject to numerous validation studies to assess reliability and responsiveness to change in therapy, including physical forms of therapy[9-11].

After familiarizing the participant to the format of the questions and the 100 mm scale, the study coordinator trained in the protocol will assess responses to the WOMAC domains.

Pain: The Visual Analog Scale (VAS) is a well-validated [138] mechanical scale used to measure pain sensation intensity evoked by nociceptive stimuli [139]. Subjects quickly and easily rate stimuli within the series by indicating level of pain on a 100 mm scale. Pain intensity is represented by the participant drawing a line on the scale indicative of pain experienced (0 = no pain, to 100 = worse pain imaginable). The VAS is anchored at the left by "no pain sensation" and at the right by "the most intense pain sensation imaginable."

No Pain	______________________________	Worst Pain Imaginable
Directions	Ask the patient to indicate on the line where the pain is in relation to the two extremes. Qualification is only approximate; for example, a midpoint mark would indicate that the pain is approximately half of the worst possible pain.	

Joint Flexibility: Joint flexibility is defined as the range of motion (ROM) allowed at the knee. The knee's ROM is measured by the number of degrees from the starting position of a segment to its position at the end of its full range of the movement. This is measured using a double-armed goniometer. A stationary arm holding a protractor is placed parallel with a stationary body segment and a movable arm moves along a moveable body segment. The pin (axis of goniometer) is placed over the joint. See figure [140].

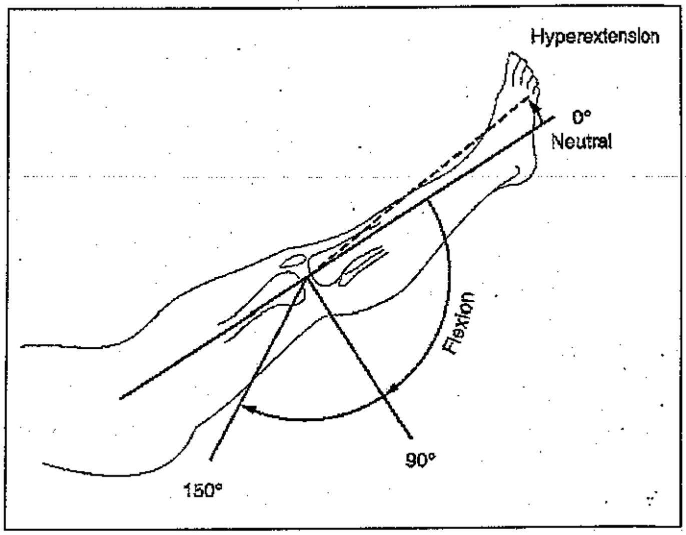

Physical function: Measured time in seconds to walk fifty (50) feet (15 m) on a level surface within the clinic facilities.

Limited Medical History: At screening and every follow-up visit participants will provide history of medication usage (prescription and over-the-counter), symptoms, duration of symptoms, date of diagnosis (screening), any change in health status and other relevant clinical information to the site study coordinator using a standardized data collection instrument.

Demographic Data: Standard demographic data (e.g. age, gender, height, weight) will be collected at baseline on all participants by the site study coordinator using a standardized data collection instrument.

Daily Concomitant Medication and Adverse Event Logs: All participants will be asked to collect and complete medication logs, indicating product used, dosage and duration of use. Study coordinators will provide calendar diaries and reminders to complete on daily any current use or change in medications and returned to the sites on a monthly basis. Questions about any experienced adverse events or changes in physical function/behavior will be collected to measure the safety of massage interventions. The study coordinator will remind participants on a regular basis through phone calls and mailings.

8.2 Laboratory Evaluations

8.2.1 Clinical Laboratory Evaluations

Not applicable.

9 ASSESSMENT OF SAFETY

The proposed protocol meets the definition of "minimal risk". "Minimal risk means that the probability and magnitude of harm or discomfort anticipated in the research are not greater in and of themselves than those ordinarily encountered in daily life or during the performance of routine physical or psychological examinations or tests" (45 CFR 46.102(h)(i)). Nevertheless, the Data Safety Monitoring Plan will encompass routine visits across sites by the principal investigators, study teams and massage therapists to ensure consistent protocol conduct and adherence to Good Clinical Practices guidelines during trial conduct.

9.1 Methods and Timing for Assessing, Recording, and Analyzing Safety Parameters

9.1.1 Adverse Events

"Adverse event" is operationally defined as any new subject complaint, including changes from the subject's medical history, which may or may not be related to the intervention. "Serious Adverse Events" are operationally defined as an experience requiring doctor or hospital visit and representing potential threat of temporary or permanent disability, or death. These include local reactions to areas receiving massage, as well as musculoskeletal injuries. Reported adverse events to massage are exceedingly rare, especially when delivered by trained massage therapists using Swedish technique (Rheumatology 2003; 42: 1101-1106).

All AEs including local and systemic reactions not meeting the criteria for "serious adverse events" should be captured on the appropriate CRF. Information to be collected includes event description, time of onset, clinician's assessment of severity, relationship to study product (assessed only by those with the training and authority to make a diagnosis, which would include MD, PA, Nurse Practitioner, DO, or DDS), and time of resolution/stabilization of the event. All AEs occurring while on study must be documented appropriately regardless of relationship. All AEs will be followed to adequate resolution.

Any medical condition that is present at the time that the patient is screened should be considered as baseline and not reported as an AE. However, if it deteriorates at any time during the study, it should be recorded as an AE.

All AEs must be graded for severity and relationship to study product.

Severity of Event: All AEs will be assessed by the clinician using a protocol defined grading system. For events not included in the protocol defined grading system, than the following guidelines will be used to quantify intensity.

- Mild: events require minimal or no treatment and do not interfere with the patient's daily activities.

- Moderate: events result in a low level of inconvenience or concern with the therapeutic measures. Moderate events may cause some interference with functioning.
- Severe: events interrupt a patient's usual daily activity and may require systemic drug therapy or other treatment. Severe events are usually incapacitating.
- Life threatening: any adverse drug experience that places the patient or subject, in the view of the investigator, at immediate risk of death from the reaction as it occurred, ie, it does not include a reaction that had it occurred in a more severe form, might have caused death.

Changes in the severity of an AE should be documented to allow an assessment of the duration of the event at each level of intensity to be performed. Adverse events characterized as intermittent require documentation of onset and duration of each episode.

Relationship to Study Products: The clinician's assessment of an AE's relationship to the test intervention is part of the documentation process, but it is not a factor in determining what is or is not reported in the study. If there is any doubt as to whether a clinical observation is an AE, the event should be reported. All AEs must have their relationship to study intervention assessed using the terms: associated or not associated. In a clinical trial, the study intervention must always be suspect. To help assess, the following guidelines are used.

- Associated – The event is temporally related to the administration of the study intervention and no other etiology explains the event.
- Not Associated – The event is temporally independent of study product and/or the event appears to be explained by another etiology.

9.1.2 Serious Adverse Events

Serious Adverse Event (SAE): An SAE is defined as an AE that meets one of the following conditions:

- Death during the period of protocol defined surveillance
- Life-threatening event (defined as a subject at immediate risk of death at the time of the event)
- An event requiring inpatient hospitalization or prolongation of existing hospitalization during the period of protocol defined surveillance
- Results in congenital anomaly or birth defect
- Results in a persistent or significant disability/incapacity
- Any other important medical event that may not result in death, be life threatening, or require hospitalization, may be considered a serious adverse experience when, based upon appropriate medical judgment, the event may jeopardize the subject and may require

medical or surgical intervention to prevent one of the outcomes listed above. Examples of such medical events include allergic bronchospasm requiring intensive treatment in an emergency room or at home, blood dyscrasias or convulsions that do not result in inpatient hospitalization, or the development of drug dependency or drug abuse.

All SAEs will be:

- recorded on the appropriate SAE CRF
- followed through resolution by a study clinician
- reviewed and evaluated by a study clinician

9.2 Reporting Procedures

Within 48 hours of a Serious Adverse Event, Adverse Event Report forms will be sent to the Principal Investigator, co-Principal Investigator, IRBs at both sites, and the program officer at NCCAM. If any authority- NCCAM, or either IRB- recommends corrective action, such as stopping the trial, the most conservative approach advised is the one that will be adopted.

9.3 Type and Duration of Follow-up of Subjects after Adverse Events

Adverse events (as described above) will be collected from monthly self-reported Adverse Event (AE) diaries maintained by study participants between scheduled clinic visits. The AE diaries collect information on the type & severity of AE, duration of AE and efforts to resolve AE. Monthly collection of these diaries will be done by the study coordinator.

This protocol meets the definition of "minimal risk" - means that the probability and magnitude of harm or discomfort anticipated in the research are not greater in and of themselves than those ordinarily encountered in daily life or during the performance of routine physical or psychological examinations or tests" (45 CFR 46.102(h)(i)). Given the extremely benign nature of the massage intervention follow-up will be limited to the principal investigator reviewing any flagged AEs and initiating any appropriate follow-up with the study participant to determine causality and steps to resolution.

9.4 Safety Oversight – Data and Safety Monitoring Plan

The NIH requires a Data and Safety Monitoring Plan (DSMP) for clinical protocols as part of the research application. Monitoring of patient data and safety is an integral component of Good Clinical Practices (GCP) and the ethical conduct of human subjects research. This is accomplished by implementation of a detailed monitoring plan to ensure data quality, protocol progress and patient safety (NIH Notice OD-00-038, June 1998 & June 2000). The DSMP is customized to the protocol and consistent with the perceived potential risks (i.e. trial, IND trial or observational protocol), size and complexity of the protocol. The plan is designed to ensure the integrity of the

data and the safety of participating research subjects. The proposed protocol meets the definition of "minimal risk". "Minimal risk means that the probability and magnitude of harm or discomfort anticipated in the research are not greater in and of themselves than those ordinarily encountered in daily life or during the performance of routine physical or psychological examinations or tests" (45 CFR 46.102(h)(i)).

The draft DSMP will encompass routine visits across sites by the principal investigators, study teams and massage therapists to ensure consistent protocol conduct and adherence to GCP guidelines during trial conduct. Adverse events (Grades 1 through 4) will be reported to the two participating institutional review boards and the sponsor for review and action.

- A member of the Delphi Expert Panel Review (Section D7) to be used at the close of the study to evaluate and identify treatment success will be asked to assist with the Data and Safety Monitoring Plan. He/She will be updated on study progress, tabulated adverse events and IRB issues during study conduct, while remaining blinded to treatment assignment.
- On a semi-annual basis investigators and staff from UMDNJ or Yale University will visit the other site to verify protocol conduct and operations consistent with the IRB-approved protocols. This will also be done with the certified massage therapists, to control for consistent interpretation and conduct of the massage therapy session across the two clinical sites. A study massage therapy session manual will be created by an independent manualization panel composed of experts in the field.
- On an annual basis a study coordinator from the PRC will visit the UMDNJ site to validate existing protocol conduct and validate a sample of the case report forms against the original source documents.

Clinical, demographic and laboratory-based data will be entered into electronic databases for management, review and analysis. Routine monitoring by the PRC data management center of case report forms collecting this information will identify and query inconsistencies, missing data and illogical choices to the clinical sites (study coordinator and PI) for clarification and correction

10 CLINICAL MONITORING

10.1 Site Monitoring Plan

Once a year, the two study teams (UMDNJ and Yale-Griffin) will meet with each other to review study progress, obstacles, issues and protocol conduct. The Yale-Griffin PRC will also send on a regular basis a staff member to conduct clinical monitoring of the UMDNJ site to insure quality and protocol conduct.

Clinical monitoring will include review of all study-related documentation, IRB correspondence, data query logs, adherence to study protocol/processes, standard operating procedures and review/refresher training of quality assurance procedures related to data collection, and participant activities.

11 STATISTICAL CONSIDERATIONS

11.1 Study Hypotheses

Specific Aim #1: **To identify the optimal practical dose and treatment regimen of an 8 week course of Swedish massage therapy for OA of the knee.**

It is crucial to identify optimal dose and treatment parameters for an intervention before proceeding to large efficacy trials. This goal will be accomplished by comparing the efficacy of the following four regimens to usual care control: 1. Thirty minutes, once a week, 2. Sixty minutes, once a week, 3. Thirty minutes, twice a week for four weeks and once a week for four weeks, and 4. Sixty minutes, twice a week for four weeks and once a week for four weeks. The regimens were chosen as practical regimens that are commonly used in massage therapy and contain a range of doses, with one higher than the pilot study and two lower doses. In addition to comparison to wait list, these regimens will be compared to each other both quantitatively and qualitatively. These regimens were designed to investigate the variables of length of individual treatment, (thirty vs. sixty minutes at once and twice a week), frequency (one vs. two times/wk at thirty and sixty minutes), and total treatment time; 240 minutes (thirty once/wk x eight wks), 480 minutes (sixty once x eight wks), or 360 minutes (thirty twice/wk x four wks and once wk x four weeks) and 720 minutes (sixty twice/wk x four wks, and sixty once/wk for four wks.

A modified Delphi Approach will determine the optimal dose/regimen(s) of the massage treatment groups listed above for OA of the knee. In addition to statistical comparisons of all outcomes of treatment arms to usual care, pair-wise comparisons between massage arms will be made. The Delphi panel (pain specialist/ rheumatologist; integrative medicine physician: licensed massage therapist) will consider the magnitude of the statistical differences as well as potential cost and convenience (length and number of visits). For example, a small difference in efficacy would not justify a four-fold increase in potential cost (e.g. 30 minutes once per week vs. 60 minutes twice/week).

We hypothesize that this combination of assessments will allow the identification of an 'optimal' dosing regimen that confers meaningful functional benefits and symptom relief, is practical for routine application into clinical practice for treatment of OA of the knee, and serves as the basis for on-going research within this line of inquiry.

Specific Aim #2: **To define the safety profile of an eight week course of Swedish massage for OA of the knee.**
Safety and tolerability will be assessed through participant self-report of symptoms or changes in symptoms since baseline (obtained from daily adverse event logs and limited history and physical) after eight weeks of massage therapy among subjects with OA of the knee. Comparison of tabulated safety events by grade (mild, moderate, severe, life-threatening) across massage treatment arms and against the wait-list control arm will help identify any unforeseen consequences of the massage regimens.

We hypothesize that all of the massage therapy dose/regimens will be safe and well tolerated, based on our pilot trial data and the safety and acceptability record of therapeutic massage, as reported in the literature.

Specific Aim #3: **To assess the duration of therapeutic effects of 8 weeks of Swedish massage on OA of the knee.**

Therapeutic effect will be measured by validated outcome measures (WOMAC Global, Visual Analog Scale pain, range of motion and time to walk 50 feet) in patients with OA of the knee at the conclusion of therapy, and 2 and 4 months post therapy (8, 16, and 24 weeks post baseline).

We hypothesize, based on our pilot trial, that decreased pain and increased function will be present at the end of treatment and persist at least two months after cessation of massage treatment. The later time point of 4 months post therapy has been added to further assess durability of therapeutic effect and is exploratory.

11.2 Sample Size Considerations

This dose-finding phase II clinical trial sample size was not based on statistical power estimation equations. At the completion of the trial, an independent three-member expert panel will be convened to review the results using a Modified Delphi Technique of consensus agreement to determine the appropriate regimen to advocate for later phase III evaluation.

- Multiple imputation techniques for analysis of incomplete data will handle missing data in order to insure adequate power and avoid biased estimates of standard errors, leading to incorrect p-values.
- Drop-out & withdrawal rate assumed at 20% cumulatively across the two clinical sites.
- An intention-to-treat analysis will be used to evaluate pre- and post-test scores to address the effects of the interventions on randomly assigned subjects whether or not they complete the study. Using the principle of last observation carried forward, missing post-test scores will be filled with the test scores that will be collected closest to the time of dropout. Secondary analyses will include tests to determine whether the interventions are effective for those subjects who actually complete the study (i.e. per-protocol analysis).
- No interim analyses planned.

Based on these issues/assumptions, final total sample size was determined to be 125 participants with randomization into five arms of 25 across the two clinical sites.

11.3 Final Analysis Plan

Subjects will be randomly assigned to a usual care or one of the four treatments. Each subject will be measured before and after treatment for the outcome measures described above. The goal of the trial is to compare the pre-post scores of the control group to the pre-post scores to the massage treatments for each outcome measure. With more than two time points of measurement in this study, two-way ANOVA using linear mixed model regression with time as repeated measure will be employed to analyze data. Correlation within subject will be modeled by incorporating a random intercept term to the model. Thus, the intercept (which corresponds to baseline value of the outcome under consideration) will be assumed to vary randomly among patients following a normal

distribution with some overall population mean and fixed variance. In addition to the effect of time on the outcome measures, other factors will be incorporated into the regression models in order to adjust for potential confounding factors (i.e., covariate imbalance not eliminated by randomization) such as demographics, clinical site, and clinical factors. Descriptive and exploratory analyses of all measured outcomes will be carried out before embarking on modeling or hypothesis testing procedures. Distributions of variables are expected to meet criteria for analysis with parametric statistics, but distributions will be assessed prior to analyses.

To ensure the comparability of study groups, ANOVA will compare baseline mean scores of all outcome measures (i.e. WOMAC, VAS, ROM, time to do 50 feet, and biomarkers) by intervention assignment. ANOVA will also assess baseline differences among subgroups by various predictor measures of interest.

Multiple imputation techniques for analysis of incomplete data will handle missing data in order to insure adequate power and avoid biased estimates of standard errors, leading to incorrect p-values. The SAS MI procedure will be used to create multiple imputed datasets. The MI procedure provides three methods for imputing missing values. Our method will depend on the type of missing data pattern. Logistic models will determine the pattern of "missingness" of observations. For monotone missing data patterns, either a parametric regression method that assumes normality or a nonparametric method with propensity scores will be used. In the regression method, a model will be fitted for each variable with missing values, with the previous variables as covariates. A new model will be simulated to impute missing values for each variable. A propensity score will be generated for each variable with missing values to indicate the probability of the observation being missing. The observations will be grouped based on propensity scores, and an approximate Bayesian bootstrap imputation will be applied to each group. For an arbitrary missing data pattern, a Markov chain Monte Carlo (MCMC) method that assumes multivariate normality will be employed. In MCMC, we will construct a Markov chain long enough for the distribution of the elements to stabilize to a common, stationary distribution. By repeatedly simulating steps of the chain, this will simulate draws from the distribution of interest. In Bayesian inference, information about unknown parameters will be expressed in the form of a posterior distribution. MCMC has been applied as a method for exploring posterior distributions in Bayesian inference. Through MCMC, we will simulate the entire joint distribution of the unknown quantities and obtain simulation-based estimates of posterior parameters that will be of interest. Assuming that the data are multivariate normally distribution, data augmentation will be applied to Bayesian inference with missing data by repeating a series of imputation and posterior steps. These steps will be iterated until the results are reliable for a multiply imputed data set [141]. Our goal will be to have the iterations converge and then to simulate an approximately independent draw of the missing values.

An **intention-to-treat analysis** will be used to evaluate pre- and post-test scores to address the effects of the interventions on randomly assigned subjects whether or not they complete the study. Using the principle of last observation carried forward, missing post-test scores will be filled with the test scores that will be collected closest to the time of dropout. Secondary analyses will include tests to determine whether the interventions are effective for those subjects who actually complete the study (i.e. per-protocol analysis).

In all analyses, a two-tailed α of less than 0.05 will be considered statistically significant. SAS software for Windows version 9.1 will be used to carry out all statistical analyses.

Specific Aim #1:<u>To identify the optimal practical dose and treatment regimen of an 8 week course of Swedish massage therapy for OA of the knee.</u> **It is crucial to identify optimal dose and treatment parameters for an intervention before proceeding to large efficacy trials. This goal will be accomplished by comparing the efficacy of the following four regimens to usual care control:** 1. Thirty minutes, once a week, 2. Sixty minutes, once a week, 3. Thirty minutes, twice a week for four weeks and once a week for four weeks, and 4. Sixty minutes, twice a week for four weeks and once a week for four weeks. The regimens were chosen as practical regimens that are commonly used in massage therapy and contain a range of doses, with one higher than the pilot study and two lower doses. In addition to comparison to wait list, these regimens will be compared to each other both quantitatively and qualitatively. These regimens were designed to investigate the variables of length of individual treatment, (thirty vs. sixty minutes at once and twice a week), frequency (one vs. two times/wk at thirty and sixty minutes), and total treatment time; 240 minutes (thirty once/wk x eight wks), 480 minutes (sixty once x eight wks), or 360 minutes (thirty twice/wk x four wks and once wk x four weeks) and 720 minutes (sixty twice/wk x four wks, and sixty once/wk for four wks.

. The regimens were chosen as practical regimens that are commonly used in massage therapy and contain a range of doses, with one higher than the pilot study and two lower doses. In addition to comparison to wait list, these regimens will be compared to each other both quantitatively and qualitatively. These regimens were designed to investigate the variables of length of individual treatment, (thirty vs. sixty minutes at once and twice a week), frequency (one vs. two times/wk at thirty and sixty minutes), and total treatment time; 240 minutes (thirty once/wk x eight wks), 480 minutes (thirty twice/wk or sixty once/wk x eight wks), or 960 minutes (sixty twice/wk x eight wks).

WOMAC: Repeated measures ANOVA using linear mixed model regression will be used to determine between-group and within-group (repeated measures ANOVA) changes in WOMAC global scores and changes in domain-specific (i.e. pain, stiffness, and functionality) WOMAC measures, controlling for time-dependent variables. In addition to the effect of time on WOMAC, other factors will be incorporated into the regression models, in order to adjust for potential confounding factors such as demographics, clinical site, severity of illness, and use of other medications.

Visual Analog Scale (VAS): Between-group and within-group changes in outcome measures from baseline to post-treatment will be compared using two-way repeated measures ANOVA. Potential confounders, such as demographics, clinical site, severity of illness and use of other medications will be controlled for in an ANCOVA model. Range of Motion (ROM): The PROC MIXED function of SAS will be used to perform repeated measures ANOVA to determine between-group changes in ROM scores, controlling for time-dependent variables. Repeated measures ANOVA will assess within-group changes in ROM scores. In addition to the effect of time on ROM scores, other factors will be incorporated into the regression models in order to adjust for potential confounding factors such as demographics, clinical site, severity of illness and use of other medications. Timed walk to 50 feet: The PROC MIXED function of SAS will be used to perform repeated measures ANOVA to assess difference between the intervention and control groups controlling for time-dependent variables. Repeated measures ANOVA will assess within-group changes in timed walk to 50 feet. Generalized linear regression will assess the effect of significant covariates on the changes in timed walk to 50 feet between the intervention and control groups. The validity of the models will be evaluated with residual analysis. Wilcoxon rank sum tests will analyze data if the validity of the models is not fulfilled.

Specific Aim #2: Safety profile: Data from the Daily Logs and limited history and physical exams conducted at time points 0, 8, 16 and 24 weeks will be tabulated by treatment assignment by complaint/sign/ symptom and severity grade (mild, moderate, and severe). 95% CIs around the event rates will be determined across treatment arms. The pilot study had one report of increased discomfort after an intervention session and ceased further study conduct and follow-up, so we anticipate few adverse events during the protocol.

Specific Aim #3: Duration of therapeutic effects: Therapeutic effect will be measured by validated outcome measures (WOMAC Global, Visual Analog Scale pain, range of motion and time to walk 50 feet) in patients with OA of the knee at the conclusion of therapy, and 2 and 4 months post therapy (8, 16, and 24 weeks post baseline). Between-group and within-group changes in outcome measures (WOMAC, VAS pain scale, range of motion and time to walk 50 feet) from baseline to post-treatment time points (8, 16 and 24 weeks) will be compared to identify duration of effect using two-way repeated measures ANOVA. Potential confounders will be controlled for in an ANCOVA model.

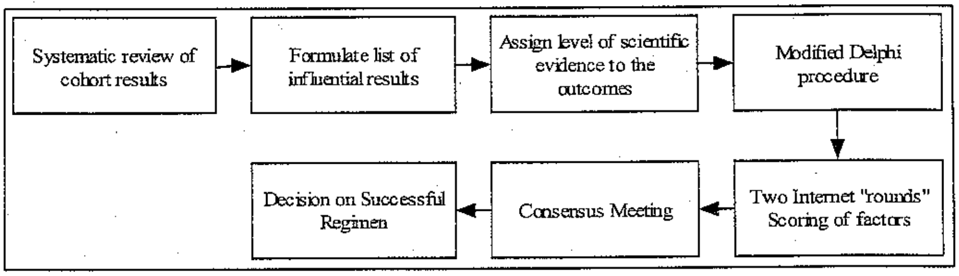

Expert Panel Review (Modified Delphi Technique): At the completion of the trial, an independent three-member expert panel will be convened to review the results and determine the appropriate regimen to advocate for later phase III evaluation. The panel will be comprised of: 1) a board-certified rheumatologist; 2) an integrative medicine internist from a major medical center with an Integrative Medicine Center and 3) a nationally certified massage therapist. All experts will have a minimum five years experience in their respective fields and provide the proper credentials to indicate their expertise and interest in the treatment of OA of the knee. The panel will be identified at the start of the project, convene to discuss the protocol prior to initiation and will be informed about study progress. Upon completion of the follow-up, results of the WOMAC, the secondary outcomes (safety, joint flexibility, physical function and biomarkers) will be summarized, analyzed and provided to the panel, initially blinded to treatment assignment. Using a modified Delphi method approach, a facilitator will stimulate initial discussion among the panel over a teleconference to solicit responses from each member, provide additional information if necessary, and assist in reaching a consensus decision of the four active massage regimens based on the study findings. Additional interaction will be done through the Internet using a secure UMDNJ community message board. Here, results will be available and the panel can respond anonymously and create a discussion thread until a consensus is reached (See Figure below). The modified Delphi Technique[136] is the most commonly used method for the production of clinical guidelines involving expert consensus. Named after the Oracle of Delphi's skills of interpretation, the process proceeds in a series of "rounds" to help reach a consensus conclusion. The expert panel will receive the dose-finding results; score the results and a final consensus review to form an opinion by repeated feedback of the unblinded dose-finding cohort results. The process has a structured format and the voting is anonymous [137].

12 SOURCE DOCUMENTS AND ACCESS TO SOURCE DATA/DOCUMENTS

Each participating site will maintain appropriate medical and research records for this trial, in compliance with ICH E6, Section 4.9 and regulatory and institutional requirements for the protection of confidentiality of subjects.

All patient information will be kept confidential. Procedures will conform to HIPAA, UMDNJ IRB, Saint Barnabas Medical Center IRB, and Griffin Hospital IRB regulations. All paper data will be maintained and secured in locked file cabinets. Unique identifiers will be used to distinguish between patient records in paper files and on the electronic spreadsheet. Copies of the CRF will be provided for use as source documents and maintained for recording data for each subject enrolled in the study.

Only individuals with appropriate OHRP certification will be permitted to handle study subject data forms. Data collection is the responsibility of the clinical trial staff at each site under the supervision of the site PI or co-investigator. During the study, the investigator must maintain complete and accurate documentation for the study.

The Yale-Griffin Prevention Research Center (PRC) will serve as the Statistical and Data Coordinating Center for this study and will be responsible for data management, quality review, analysis, and reporting of the study data. The PI and co-investigator at UMDNJ will send data materials on a monthly basis to the PRC to scan and analyze. Photocopies of all data sent will be kept on site at UMDNJ.

13 QUALITY CONTROL AND QUALITY ASSURANCE

Study Coordination: The study teams will meet in the first quarter of the project to develop all the appropriate tools and processes for study conduct. This includes the protocol, the consent forms, the case report forms, the instruments to be used, the manual of operations and all other standard operating procedures. The Manualization Panel will convene as described to create a manual for the massage therapy sessions. Site study coordinators will be MPH trained, experienced in the conduct of clinical research, and knowledgeable of Good Clinical Practices guidelines in the conduct of clinical trials. The study coordinators will work with the study investigators at the implementation of the protocols, which includes volunteer screening; assistance with advertising; providing participant informed consent; and assisting participants with completing the outcome instruments; reminders to participants about daily logs; and work with the PRC for data collection and query resolution. To maintain study coordinator blinding of treatment assignment, hospital staff at the Siegler Center and the Integrative Medicine Center at Griffin Hospital will arrange massage session appointments, provide appointment reminders, and contact the PRC for participant randomization. In addition, all evaluations of participants by the study coordinators will occur at separate location from the sites of the massage intervention.

All source documents and laboratory reports must be reviewed by the clinical team and data entry staff, who will ensure that they are accurate and complete. Adverse events must be graded, assessed for severity and causality, and reviewed by the site PI or designee.
Data collection is the responsibility of the clinical trial staff at each site under the supervision of the site PI or co-investigator. During the study, the investigator must maintain complete and accurate documentation for the study.

The Yale-Griffin Prevention Research Center (PRC) will serve as the Statistical and Data Coordinating Center for this study and will be responsible for data management, quality review, analysis, and reporting of the study data. The PI and co-investigator at UMDNJ will send data materials on a monthly basis to the PRC to scan and analyze. Photocopies of all data sent will be kept on site at UMDNJ.

Once a year, the two study teams (UMDNJ and Yale-Griffin) will meet with each other to review study progress, obstacles, issues and protocol conduct. The PRC will also send on a regular basis a staff member to conduct clinical monitoring of the UMDNJ site to insure quality and protocol conduct.

All sites will be trained in standard study management procedures as well as the designated massage protocol by the study coordinator and massage therapists at the Siegler Center. The study coordinator will conduct an annual site visit at the Yale center to for quality assurance of all procedures. The site visits will include training on inclusion/exclusion criteria, consent, retention, instrument use, blinding, massage technique, and other logistics. Sites will be assessed to ensure strict adherence to study protocol. All tools will be provided by UMDNJ based on material successfully used in the published pilot study.

Site coordinators will participate in monthly conference calls hosted by the Institute for Complementary and Alternative Medicine to discuss trial logistics and progress. These will be attended by the Principal Investigator, Data Manager, and Study Coordinator. On a semi-annual basis, study teams from each site will visit the other site to discuss study logistics, performance and provide quality assurance in protocol conduct. Prior to conduct, massage therapists from each site will meet with study staff to review protocol conduct, massage techniques described in detail in the Massage Manual and come to consensus any therapeutic massage issues prior to protocol implementation.

14 ETHICS/PROTECTION OF HUMAN SUBJECTS

14.1 Ethical Standard

The investigator will ensure that this study is conducted in full conformity with the principles set forth in The Belmont Report: Ethical Principles and Guidelines for the Protection of Human Subjects of Research of the US National Commission for the Protection of Human Subjects of Biomedical and Behavioral Research (April 18, 1979) and codified in 45 CFR Part 46 and/or the ICH E6; 62 Federal Regulations 25691 (1997).

14.2 Institutional Review Board

Each participating institution must provide for the review and approval of this protocol and the associated informed consent documents and recruitment material by an appropriate independent ethics committee (IEC) or IRB registered with the OHRP. Any amendments to the protocol or consent materials must also be approved before they are placed into use.

Performance Sites	OHRP Assurance Numbers	Expiration
UMDNJ – School of Health Related Professions, Newark, NJ	FWA00000036	April 24, 2011
St. Barnabas Medical Center, Livingston, NJ	FWA00003433	September 22, 2008
Yale School of Medicine, New Haven, CT	FWA00002571	November 16, 2008
Griffin Hospital, Derby, CT	FWA00000037	November 22, 2009

14.3 Informed Consent Process

A written informed consent form approved by each site's Institutional Review Board (IRB) will be used to obtain participant consent prior to clinical screening. Extensive discussion of risks and possible benefits of this therapy will be provided to the subjects and their families. Consent forms describing in detail the massage interventions, study procedures, and risks are given to the subject and written documentation of informed consent is required prior to starting intervention. Upon reviewing the document, the investigator will explain the research study to the subject and answer any questions that may arise. The subjects will sign the informed consent document prior to any procedures being done specifically for the study. Informed consent will be obtained by the study coordinator at each site. All participants will be informed of the option of not participating, and of stopping at anytime during the study. A copy of the informed consent document will be given to the subjects for their records.

The rights and welfare of the subjects will be protected by emphasizing to them that the quality of their medical care will not be adversely affected if they decline to participate in this study. Consent procedures will conform to HIPAA requirements and human subjects protection requirements of the participating institutional review boards.

14.4 Exclusion of Women, Minorities, and Children (Special Populations)

Inclusion of Women: Eligible female volunteers will be approached for participation in this trial at both participating clinical sites, as osteoarthritis of the knee is highly prevalent in women.

Inclusion of Minorities: Eligible minority volunteers will be approached for participation in this trial at both participating clinical sites, as osteoarthritis of the knee is prevalent in all races and ethnic groups.

Inclusion of Children: Children will not be approached to participate in this trial as osteoarthritis of the knee is a chronic condition typically seen in the elderly population [5].

In the earlier study, conducted solely at St. Barnabas Medical Center, approximately 80% of the cohort was female and 14% were non-white in ethnicity/race. Based on practice profile we anticipate a similar mix of participants (reflective of osteoarthritis epidemiology) at the Integrative Medicine Center at Griffin Hospital.

14.5 Subject Confidentiality

Subject confidentiality is strictly held in trust by the participating investigators, their staff, and the sponsor(s) and their agents.

All data obtained during the course of the study will be stored both electronically and in hard copy. The study coordinator will be responsible for ensuring completion of all study forms and collection of all data materials. Survey and personal data will be entered into an encrypted electronic spreadsheet by a dedicated data manager and/or the study coordinator at each site to help coordinate massage session and follow-up schedules. All data for the study will be entered in Cardiff Teleform-compatible scannable forms by a dedicated data manager. Once all entries have been made, the raw data files will be used to create SAS datasets.

To protect the privacy of patients, all patient information will be kept confidential. Procedures will conform to HIPAA, UMDNJ IRB, Saint Barnabas Medical Center IRB, Yale/Griffin Hospital IRB, and Yale University HIC regulations. All paper data will be maintained and secured in locked file cabinets. Unique identifiers will be used to distinguish between patient records in paper files and on the electronic spreadsheet. Only individuals with appropriate OHRP certification will be permitted to handle study subject data forms.

The study monitor or other authorized representatives of the sponsor may inspect all documents and records required to be maintained by the investigator. The clinical study site will permit access to such records.

14.6 Future Use of Stored Specimens

Not Applicable

15 DATA HANDLING AND RECORD KEEPING

The PI and co-investigators are responsible for ensuring the accuracy, completeness, legibility, and timeliness of the data reported. All source documents will be completed in a neat, legible manner to ensure accurate interpretation of data. Black ink is required to ensure clarity of reproduced copies. When making changes or corrections, the original entry will be crossed out with a single line, initialed and dated.

Copies of the CRF will be provided for use as source documents and maintained for recording data for each subject enrolled in the study.

DMID and/or it's designee will provide guidance to investigators on making corrections to the source documents and CRF.

All patient information will be kept confidential. Procedures will conform to HIPAA, UMDNJ IRB, Saint Barnabas Medical Center IRB, and Griffin Hospital IRB regulations. All paper data will be maintained and secured in locked file cabinets. Unique identifiers will be used to distinguish between patient records in paper files and on the electronic spreadsheet. Only individuals with appropriate OHRP certification will be permitted to handle study subject data forms.

15.1 Data Management Responsibilities

All source documents and laboratory reports must be reviewed by the clinical team and data entry staff, who will ensure that they are accurate and complete. Adverse events must be graded, assessed for severity and causality, and reviewed by the site PI or designee.

Data collection is the responsibility of the clinical trial staff at each site under the supervision of the site PI or co-investigator. During the study, the investigator must maintain complete and accurate documentation for the study.

The Yale-Griffin Prevention Research Center (PRC) will serve as the Statistical and Data Coordinating Center for this study and will be responsible for data management, quality review, analysis, and reporting of the study data. The PI and co-investigator at UMDNJ will send data materials on a monthly basis to the PRC to scan and analyze. Photocopies of all data sent will be kept on site at UMDNJ.

15.2 Data Capture Methods

All data obtained during the course of the study will be stored both electronically and in hard copy. Survey and personal data will be entered into an encrypted electronic spreadsheet by a dedicated data manager and/or the study coordinator at each site to help coordinate massage session and follow-up schedules. All data for the study will be entered in Cardiff Teleform-compatible scannable forms by a dedicated data manager. Once all entries have been made, the raw data files will be used to create SAS datasets.

15.3 Types of Data

Data for this study will include self-reported safety/adverse events, demographic, medical history information, concomitant medications and outcome measures (eg, self-report questionnaire responses to the WOMAC scales, VAS pain scale, and measurements of range of motion and time to walk 50 feet).

15.4 Timing/Reports

Data for this study will include self-reported safety/adverse events, demographic, medical history information, concomitant medications and outcome measures (eg, self-report questionnaire responses to the WOMAC scales, VAS pain scale, and measurements of range of motion and time to walk 50 feet).

15.5 Study Records Retention

Study documents will be retained for five(5) years after the completion of the proposed clinical trial – as per required by the UMDNJ Institutional Review Board.

Protocol Deviations

A protocol deviation is any noncompliance with the clinical trial protocol, Good Clinical Practice (GCP), or Manual of Procedures requirements. The noncompliance may be either on the part of the subject, the investigator, or the study site staff. As a result of deviations, corrective actions are to be developed by the site and implemented promptly.
These practices are consistent with ICH E6:

- Compliance with Protocol, sections 4.5.1, 4.5.2, and 4.5.3
- Quality Assurance and Quality Control, section 5.1.1
- 5.20 Noncompliance, sections 5.20.1, and 5.20.2.

It is the responsibility of the site to use continuous vigilance to identify and report deviations within 5 working days of identification of the protocol deviation, or within 5 working days of the scheduled protocol-required activity.

16 PUBLICATION POLICY

Following completion of the study, the investigator is expected to publish the results of this research in a scientific journal. The International Committee of Medical Journal Editors (ICMJE) member journals have adopted a trials-registration policy as a condition for publication. This policy requires that all clinical trials be registered in a public trials registry such as ClinicalTrials.gov*, which is sponsored by the National Library of Medicine. Other biomedical journals are considering adopting similar policies. It is the responsibility of DMID to register this trial in an acceptable registry. Any clinical trial starting enrollment after 01 July 2005 must be registered on or before patient enrollment. For trials that began enrollment prior to this date, the ICMJE member journals will require registration by 13 September 2005, before considering the results of the trial for publication.

The ICMJE defines a clinical trial as any research project that prospectively assigns human subjects to intervention or comparison groups to study the cause-and-effect relationship between a medical intervention and a health outcome. Studies designed for other purposes, such as to study pharmacokinetics or major toxicity (eg, Phase I trials), would be exempt from this policy.

*Journal Citation:

De Angelis C, Drazen JM, Frizelle FA, Haug C, Hoey J, Horton R, et al. Clinical trial registration: a statement from the International Committee of Medical Journal Editors. N Engl J Med. 2004;351:1250-1.

17 LITERATURE REFERENCES

1. Praemer, A., S. Furner, and D. Rice, Musculoskeletal Conditions in the United States. . American Academy of Orthopaedic Surgeons. 1999, Rosemont, IL.
2. Hinton, R., R.L. Moody, A.W. Davis, and S. Thomas, Osteoarthristis: Diagnosis and Therapeutic Considerations. American Family Physicans, 2002. 65(5): p. 841-848.
3. Lawrence, R., Helmick CG, Arnett FC, Deyo RA, Felson DT, Giannini EH, Heyse SP, Hirsch R, Hochberg MC, Hunder GG, Liang MH, Pillemer SR, Steen VD, and W. F., Estimates of the prevalence of arthritis and selected musculoskeletal disorders in the United States. Arthritis Rheum, 1998. 41: p. 778-799.
4. Matchaba, P., Gitton X, Krammer G, Ehrsam E, Sloan VS, Mellein B, Hoexter G, Orloff J, and G. JJ., Cardiovascular safety of lumiracoxib: A meta-analysis of all randomized controlled trials ^{3} 1 week and up to 1 year in duration of patients with osteoarthritis and rheumatoid arthritis. Cardiovascular safety of lumiracoxib: A meta-analysis of all randomized controlled trials ^{3} 1 week and up to 1 year in duration of patients with osteoarthritis and rheumatoid arthritis. Clin Ther, 2005. 27: p. 1196-1214.
5. Felson, D., An update on the pathogenesis and epidemiology of osteoarthritis. Radiol Clin North Am, 2004. 42: p. 1-9.
6. Naesdal, J. and B. K., NSAID-associated adverse effects and acid control aids to prevent them: A review of current treatment options. Drug Saf, 2006. 29: p. 119-132.
7. Felson, D., R. Lawrence, M. Hochberg, and etal., Osteoarthritis:new insights, II: treatment approaches. Ann Intern Med, 2000. 133: p. 726-737.
8. Perlman, A.I., A. Sabina, A.L. Williams, V.Y. Njike, and D.L. Katz, Massage therapy for osteoarthritis of the knee: a randomized controlled trial. Arch Intern Med, 2006. 166(22): p. 2533-8.
9. Bellamy, N., The WOMAC knee and hip osteoarthritis indices: Development, validation, globalization and influence on the development of the AUSCAN hand osteoarthritis indices. Clin Exp Rheumatol, 2005. 23(5 suppl 39): p. S148-153.
10. Bellamy, N., W. Buchanan, C. Goldsmith, J. Campbell, and L. Stitt, Validation study of WOMAC: a health status instrument for measuring clinically important patient relevant outcomes to antirheumatic drug therapy in patients with osteoarthritis of the hip or knee J Rheumatology, 1988. 15: p. 1833-1840.
11. McConnell, S., P. Kolopack, and A. Davis, The Western Ontario and McMaster Universities Osteoarthritis Index (WOMAC): a review of its utility and measurement properties. Arthritis Rheum, 2001. 45(5): p. 453-461.
12. Elders, M., The increasing impact of arthritis on public health. J Rheumatol., 2000. 60(suppl): p. 6-8.
13. Arden, N. and M. Nevitt, Osteoarthritis:epidemiology. Best Pract Res Clin Rheumatol, 2006. 20: p. 3-25.

14. Gelber, A.C., M.C. Hochberg, L.A. Mead, N.Y. Wang, F.M. Wigley, and M.J. Klag, Joint injury in young adults and risk for subsequent knee and hip osteoarthritis. Ann Intern Med, 2000. 133(5): p. 321-8.
15. Roos, E.M., Joint injury causes knee osteoarthritis in young adults. Curr Opin Rheumatol, 2005. 17(2): p. 195-200.
16. Peyron, J., Osteoarthritis: The epidemiologic viewpoint Clin Ortho Rel Res, 1986. 213: p. 139-19.
17. Adams, P. and A. Hardy, Current Estimates From the National Health Interview Survey, 1988. Vital Health Stat Series 10 (173). 1989, National Center for Health Statistics.
18. Kelsey, J. and M. Hochberg, Epidemiology of chronic musculoskeletal disorders Annu Rev Public Health 1988: p. 379-401
19. Kato, T., Y. Xiang, H. Nakamura, and K. Nishioka, Neoantigens in osteoarthritic cartilage. Curr Opinion Rheumatol, 2004. 16: p. 604-608.
20. Fisher, N. and D. Pendergast, Reduced muscle function in patients with osteoarthritis. Scand J Rehab Med, 1997. 29: p. 213-221.
21. Messier, S., R. Loeser, J. Hoover, E. Semble, and C. Wse, Osteoarthritis of the knee:Effects on gait, strength, and flexibility. Arch Phys Med Rehabil 1992. 73: p. 29-36.
22. Gibofsky, A. and R.L. Barkin, Chronic pain of osteoarthritis: considerations for selecting an extended-release opioid analgesic. Am J Ther, 2008. 15(3): p. 241-55.
23. Jakobsson, U. and I.R. Hallberg, Quality of life among older adults with osteoarthritis: an explorative study. J Gerontol Nurs, 2006. 32(8): p. 51-60.
24. Wilcox, S., G.A. Brenes, D. Levine, M.A. Sevick, S.A. Shumaker, and T. Craven, Factors related to sleep disturbance in older adults experiencing knee pain or knee pain with radiographic evidence of knee osteoarthritis. J Am Geriatr Soc, 2000. 48(10): p. 1241-51.
25. Hunter, D. and D. Felson, Osteoarthritis. British Medical Journal, 2006. 332: p. 639-642
26. MacLean, C.H., Costs attributable to osteoarthritis. The Journal of rheumatology. Supplement, 1998. 25(11): p. 2213-8.
27. Maetzel, A., Annals of the rheumatic diseases. Ann Rheum Dis., 2004 63(4): p. 395-401.
28. Lanes, S.F., Resource utilization and cost of care for rheumatoid arthritis an osteoarthritis in a managed care setting: the importance of drug and surgery costs. Arthritis Rheum, 1997. 40(8): p. 1475-81.
29. Coupe, V.M.H., The cost effectiveness of behavioural graded activity in patients with osteoarthritis of hip and/or knee. Annals of the rheumatic diseases, 2007. 66(2): p. 215-21.
30. SooHoo, N.F., Cost-effectiveness analysis of total ankle arthroplasty. The Journal of bone and joint surgery, 2004 American p. 2446-55.
31. Lapsley, H.M., Living with osteoarthritis: patient expenditures, health status, and social impact. Arthritis and rheumatism, 2001 45(3): p. 301-6.
32. Gupta, S., The economic burden of disabling hip and knee osteoarthritis (OA) from the perspective of individuals living with this condition. Rheumatology (Oxford, England), 2005 44(12): p. 1531-7.
33. Ricci, J.A., W.F. Stewart, E. Chee, C. Leotta, K. Foley, and M.C. Hochberg, Pain exacerbation as a major source of lost productive time in US workers with arthritis. Arthritis Rheum, 2005. 53(5): p. 673-81.

34. Bailey, A. and J. Mansell, Do subchondral bone changes exacerbate or precede articular cartilage destruction in osteoarthritis of the elderly? Gerontology, 1997. 43: p. 296-304.
35. Carlson, C.S., R.F. Loesser, C.B. Purser, J.F. Gardin, and C.P. Jerome, Osteoarthritis in cyanomolgus macques II. Effects of age, gender and subchondral bone thickness on the severity of the disease. J. Bone Miner, 1996. 11: p. 1209-1217.
36. Bayliss, M., C. Davidson, S. Woodhouse, and D. Osborne, Chondroitin sulphate in human joint varies with age, zone, and topography. Acta Orthop Scand Suppl, 1995. 266: p. 22-25.
37. Slater, R., M. Bayliss, P. Lachiewicz, D. Visco, and B. Caterson, Monoclonal antibodies that detect biochemical markers of arthritis in humans. Arthritis Rheum, 1995. 38: p. 655-659.
38. Radin, E.L., R.M. Rose, and yd, Role of subchondral bone in the initation and progression of cartilage damage. . Clin. Orthop. Rel. Res, 1986. 213: p. 34-40.
39. Fiechtner, J.J. and R.R. Brodeur, Manual and manipulation techniques for rheumatic disease. Medical Clinics of North America, 2002. 86(1): p. 91-103.
40. Roit, I., J. Brostoff, and D. Male, Immunology. 6th ed. 2001, London.
41. Son, D.J., J.W. Lee, Y.H. Lee, H.S. Song, C.K. Lee, J.T. Hong, D.J. Son, J.W. Lee, Y.H. Lee, H.S. Song, C.K. Lee, and J.T. Hong, Therapeutic application of anti-arthritis, pain-releasing, and anti-cancer effects of bee venom and its constituent compounds. Pharmacology & Therapeutics, 2007. 115(2): p. 246-70.
42. De Jongh, R.F., K.C. Vissers, T.F. Meert, L.H. Booij, C.S. De Deyne, and R.J. Heylen, The role of interleukin-6 in nociception and pain. Anesth Analg, 2003. 96(4): p. 1096-103, table of contents.
43. De Jongh, R.F., K.C. Vissers, L.H. Booij, K.L. De Jongh, P. Vincken, and T.F. Meert, Interleukin-6 and perioperative thermoregulation and HPA-axis activation. Cytokine, 2003. 21(5): p. 248-56.
44. Pace, M.C., L. Mazzariello, M.B. Passavanti, P. Sansone, M. Barbarisi, and C. Aurilio, Neurobiology of pain. Journal of Cellular Physiology, 2006. 209(1): p. 8-12.
45. Gibofsky, A. and R. Barkin, Chronic Pain of Osteoarthritis: Considerations for Selecting an Extended - Release Opioid Analgesic. American Journal of Therapeutics, 2008. 15(3): p. 241-255.
46. Katz, W. and R. Barkin, Dilemmas in Chronic/Persistent Pain Management. American Journal of Therapeutics, 2008. 15(3): p. 256-264.
47. Goldring, S.R. and M.B. Goldring, The role of cytokines in cartilage matrix degeneration in osteoarthritis. Clinical Orthopaedics & Related Research, 2004(427 Suppl): p. S27-36.
48. Nakamura, H., S. Yoshino, T. Kato, J. Tsuruha, and K. Nishioka, T-cell mediated inflammatory pathway in osteoarthritis. Osteoarthritis & Cartilage, 1999. 7(4): p. 401-2.
49. Kobayashi, M., G.R. Squires, A. Mousa, M. Tanzer, D.J. Zukor, J. Antoniou, U. Feige, and A.R. Poole, Role of interleukin-1 and tumor necrosis factor alpha in matrix degradation of human osteoarthritic cartilage. Arthritis & Rheumatism, 2005. 52(1): p. 128-35.
50. Abramson, S.B. and Y. Yazici, Biologics in development for rheumatoid arthritis: relevance to osteoarthritis. Advanced Drug Delivery Reviews, 2006. 58(2): p. 212-25.
51. Abramson, S.B., A.R. Amin, R.M. Clancy, and M. Attur, The role of nitric oxide in tissue destruction. Best Practice & Research in Clinical Rheumatology, 2001. 15(5): p. 831-45.

52. Abramson, S.B., M. Attur, A.R. Amin, and R. Clancy, Nitric oxide and inflammatory mediators in the perpetuation of osteoarthritis. Current Rheumatology Reports, 2001. 3(6): p. 535-41.
53. Alaaeddine, N., J.A. Di Battista, J.P. Pelletier, K. Kiansa, J.M. Cloutier, and J. Martel-Pelletier, Inhibition of tumor necrosis factor alpha-induced prostaglandin E2 production by the antiinflammatory cytokines interleukin-4, interleukin-10, and interleukin-13 in osteoarthritic synovial fibroblasts: distinct targeting in the signaling pathways. Arthritis & Rheumatism, 1999. 42(4): p. 710-8.
54. Jikko, A., T. Wakisaka, M. Iwamoto, H. Hiranuma, Y. Kato, T. Maeda, M. Fujishita, and H. Fuchihata, Effects of interleukin-6 on proliferation and proteoglycan metabolism in articular chondrocyte cultures. Cell Biology International, 1998. 22(9-10): p. 615-21.
55. Cicuttini, F., J. Baker, and T. Spector, The association of obesity with osteoarthritis of the hand and knee in women. J Rheumatol, 1996. 23: p. 1221-1226.
56. Huebner, J.L. and V.B. Kraus, Assessment of the utility of biomarkers of osteoarthritis in the guinea pig. Osteoarthritis Cartilage, 2006. 14(9): p. 923-30.
57. Ameye, L.G. and M.F. Young, Animal models of osteoarthritis: lessons learned while seeking the "Holy Grail". Curr Opin Rheumatol, 2006. 18(5): p. 537-47.
58. Fantuzzi, G., Adipose tissue, adipokines, and inflammation. J Allergy Clin Immunol, 2005. 115: p. 911-919.
59. Gould Fogerite, S. and G. Goldberg, Overview of Mind-Body Therapies, in Complemplementary Therapies for Physical Therapy: A Clinical Decision Making Approach, J. Deutch and E. Anderson, Editors. 2008, Saunders, Elsevier: St. Loius. p. 84-120.
60. Katz, W.A. and R.L. Barkin, Dilemmas in chronic/persistent pain management. Am J Ther, 2008. 15(3): p. 256-64.
61. Siddall, P.J. and M.J. Cousins, Persistent pain as a disease entity: implications for clinical management. Anesth Analg, 2004. 99(2): p. 510-20, table of contents.
62. Brookoff, D., Chronic pain: 1. A new disease? Hosp Pract (Minneap), 2000. 35(7): p. 45-52, 59.
63. Rygh, L.J., F. Svendsen, A. Fiska, F. Haugan, K. Hole, A. Tjolsen, L.J. Rygh, F. Svendsen, A. Fiska, F. Haugan, K. Hole, and A. Tjolsen, Long-term potentiation in spinal nociceptive systems--how acute pain may become chronic. Psychoneuroendocrinology, 2005. 30(10): p. 959-64.
64. Gilron, I., C.P. Watson, C.M. Cahill, and D.E. Moulin, Neuropathic pain: a practical guide for the clinician. CMAJ, 2006. 175(3): p. 265-75.
65. Vikman, K.S., P.J. Siddall, and A.W. Duggan, Increased responsiveness of rat dorsal horn neurons in vivo following prolonged intrathecal exposure to interferon-gamma. Neuroscience, 2005. 135(3): p. 969-77.
66. Ma, W., R. Quirion, W. Ma, and R. Quirion, Targeting invading macrophage-derived PGE2, IL-6 and calcitonin gene-related peptide in injured nerve to treat neuropathic pain. Expert Opinion on Therapeutic Targets, 2006. 10(4): p. 533-46.
67. Knotkova, H., M. Pappagallo, and A. Szallasi, Capsaicin (TRPV1 Agonist) therapy for pain relief: farewell or revival? Clin J Pain, 2008. 24(2): p. 142-54.

68. Park, H.J., D.J. Son, C.W. Lee, M.S. Choi, U.S. Lee, H.S. Song, J.M. Lee, and J.T. Hong, Melittin inhibits inflammatory target gene expression and mediator generation via interaction with IkappaB kinase. Biochem Pharmacol, 2007. 73(2): p. 237-47.
69. Kong, S., T. Stabler, L. Criscione, J. Elliott, and V.B. Kraus, Diurnal variation of serum and urine biomarkers in patients with radiographic knee arthritis. Arthritis and Rheumatism, 2006. 54: p. 2496-2504.
70. Kraus, V.B., Do biochemical markers have a role in osteoarthritis diagnosis and treatment? Best Prac Res Clin Rheum, 2006. 20: p. 69-80.
71. Nakamura, H., M. Tanaka, K. Masuko-Hongo, K. Yudoh, T. Kato, M. Beppu, and K. Nishioka, Enhanced production of MMP-1, MMP-3, MMP-13, and RANTES by interaction of chondrocytes with autologous T cells. Rheumatol Int, 2006. 26(11): p. 984-90.
72. Spector, T., D. Hart, D. Nandra, D. Doyle, N. Mackilop, and J. Gallimore, Low-level increases in serum C - reactive protein are present in early osteoarthritis of the knee and predict progressive disease. Ann Rehum 1997. 40: p. 723-7.
73. Sturmer, T., H. Brenner, W. Koeing, and K. Gunther, Severity and extent of osteoarthritis and low grade systemic inflammation as assessed by high sensitivity C reative protein. Ann Rheum Dis, 2004. 63: p. 200-5.
74. Sharma, L., M. Ionescu, and J. Song, The ratio of collagen breakdown synthesis and its realtionship with the progression of knee osteoarthritis. Arthritis and Rheumatism, 2004. 50 (supplement 9): p. 642 [see also 282].
75. Garnero, P., X. Ayral, and J. Rousseau, Uncoupling of type II collagen synthesis and degradation predicts progression of joint damage in patients with knee osteoarthritis. Arthritis and Rheumatism, 2002. 46(10): p. 2613-2624.
76. Chrosous, G., The hypothalamic-pituitary-adrenal axis and immune-meidated inflammatin. N Engl J Med, 1995. 332: p. 1351-1360.
77. Jo, H., H.J. Ahn, E.M. Kim, H.J. Kim, S.C. Seong, I. Lee, and M.C. Lee, Effects of dehydroepiandrosterone on articular cartilage during the development of osteoarthritis. Arthritis & Rheumatism, 2004. 50(8): p. 2531-8.
78. Cruess, D., M. Antoni, M. Kumar, G. Ironson, McCabe, J. Fernandez, M. Fletcher, and N. Scheniderman, Cognitive-behavior stress management buffers decreases n Dehydroepiandrosterone sulfate (DHEA-S) and increases in the cortisol/DHEA-S ratio and reduces mood disturbance and perceived stress among HIV- seropositive men. Psycholneuroendocrinology, 1999. 24: p. 537-549.
79. Carlson, L., M. Speca, K. Patel, and E. Goodey, Mindfullness-based stress reduction in relation to quality of life, mood, symptoms of stress and levels of cortisol, DHEAS, and melatonin in breast cancer patients. Psychoneuroendocrinology, 2002. 29: p. 448-474.
80. Towheed, T.E., L. Maxwell, M.G. Judd, M. Catton, M.C. Hochberg, and G. Wells, Acetaminophen for osteoarthritis. Cochrane Database Syst Rev, 2006(1): p. CD004257.
81. Crofford, L.J., COX-1 and COX-2 tissue expression: implications and predictions. J Rheumatol Suppl, 1997. 49: p. 15-9.
82. Ernst, E., Complementary and alternative medicine for pain management in rheumatic disease Curr Opin Rheum 2002. 14: p. 58-62.

83. National Center for Complementary and Alternative Medicine website. 5-12-07 [cited; Available from: http://nccam.nih.gov.
84. Diehl, D.L. and D. Eisenberg, Complementary and alternative medicine (CAM): epidemiology and implications for research. Progress in Brain Research, 2000. 122: p. 445-55.
85. Conboy, L., S. Patel, T.J. Kaptchuk, B. Gottlieb, D. Eisenberg, and D. Acevedo-Garcia, Sociodemographic determinants of the utilization of specific types of complementary and alternative medicine: an analysis based on a nationally representative survey sample. Journal of Alternative & Complementary Medicine, 2005. 11(6): p. 977-94.
86. Eisenberg, D.M., R.B. Davis, S.L. Ettner, S. Appel, S. Wilkey, M. Van Rompay, and R.C. Kessler, Trends in alternative medicine use in the United States, 1990-1997: results of a follow-up national survey.[see comment]. JAMA, 1998. 280(18): p. 1569-75.
87. Eisenberg, D.M., R.C. Kessler, C. Foster, F.E. Norlock, D.R. Calkins, and T.L. Delbanco, Unconventional medicine in the United States: Prevalence, costs, and patterns of use N Engl J Med, 1993. 328: p. 246-252.
88. Foster, D.F., R.S. Phillips, M.B. Hamel, and D.M. Eisenberg, Alternative medicine use in older Americans.[see comment]. Journal of the American Geriatrics Society, 2000. 48(12): p. 1560-5.
89. Roberts, D., Alternative therapies for arthritis treatment: part 2. Holistic Nursing Practice, 2004. 18(3): p. 167-74.
90. Astin, J.A., Mind-body therapies for the management of pain. Clinical Journal of Pain, 2004. 20(1): p. 27-32.
91. Perlman, A. and Meng, C, "Rheumatology," in Complementary and Alternative Medicine in Rehabilitation, E. Leskowitz., Editor. 2003, Harcourt Heath Sciences, : St. Louis, MO. p. 352-362.
92. Perlman, A. and Spierer, M, "Osteoarthritis," in Integrative Medicine, D. Rakel., Editor. 2003, W.B. Saunders Co.: Orlando, FL. . p. 414-422.
93. Perlman, A. and Weisman. R, Own Your Own Health - The Best of Alternative and Conventional Medicine: Pain. 2006, Deerfield Beach, FL: Health Communications, Inc.
94. Wolsko, P.M., D.M. Eisenberg, L.S. Simon, R.B. Davis, J. Walleczek, M. Mayo-Smith, T.J. Kaptchuk, and R.S. Phillips, Double-blind placebo-controlled trial of static magnets for the treatment of osteoarthritis of the knee: results of a pilot study. Alternative Therapies in Health & Medicine, 2004. 10(2): p. 36-43.
95. Hrobjartsson, A., The uncontrollable placebo effect. Eur J. Clin. Pharmacol., 1996. 50(5): p. 345-8.
96. Tappan, F., Healing massage techniques: Holistic, classic and emerging methods. 1988, Norwalk: Appleton and Lange.
97. Nayak, S., R. Matheis, S. Agostinelli, and S. Shifleft, The use of complementary and alternative therapies for chronic pain following spinal cord injury: A pilot survey J Spinal Cord Med. , 2001. 24((1)): p. 54-62.
98. Schaible, H.-G. and B. Grubb, Afferent and spinal mechanisms of joint pain. Pain, 1993. 55: p. 5-54.

99. Huijing, P.A., Muscle as a collagen fiber reinforced composite: a review of force transmission in muscle and whole limb. Fascia Research, ed. T. Findley and R. Schleip. 2007, Munchen, Germany: Elsevier. 90-107.

100. Stahl, V.A., J.S. Gottschall, and T.R. Nichols, A biomechanical analysis of the crural fascia in the feline hindlimb during locomotion and intra-muscular stimulation. Fascia Research, ed. T. Findley and R. Schleip. 2007, Munchen, Germany: Elsevier.

101. Field, T., Massage therapy. Med Clin North Am, 2002. 86(1): p. 163-71.

102. Field, T., M. Diego, C. Cullen, M. Hernandez-Reif, W. Sunshine, and S. Douglas, Fibromyalgia pain and substance P decrease and sleep improves after massage therapy. J Clin Rheumatol, 2002. 8(2): p. 72-6.

103. Diego, M.A., T. Field, and M. Hernandez-Reif, Vagal activity, gastric motility, and weight gain in massaged preterm neonates. J Pediatr, 2005. 147(1): p. 50-5.

104. Wilkie, D., J. Kampbell, S. Cutshall, H. Halabisky, H. Harmon, L. Johnson, L. Weinacht, and M. Rake-Marona, Effects of massage on pain intensity, analgesics and quality of life in patients with cancer pain: a pilot study of a randomized clinical trial conducted within hospice care delivery. Hosp J., 2000. 15((3)): p. 31-53.

105. Bardia, A., D.L. Barton, L.J. Prokop, B.A. Bauer, and T.J. Moynihan, Efficacy of complementary and alternative medicine therapies in relieving cancer pain: a systematic review. Journal of Clinical Oncology, 2006. 24(34): p. 5457-64.

106. Cherkin, C., K. Sherman, R. Devo, and P. Shekelle, A review of the evidence for the effectiveness, safety, and cost of acupuncture, massage therapy, and spinal manipulation for back pain. Ann Intern Med. , 2003 138 (11): p. 898-906

107. Preyde, M., Effectiveness of massage therapy for subacute low-back pain: A randomized controlled trial CMAJ 2000. 162(13): p. 1815-1820.

108. Hernandez-Reif, M., T. Field, J. Krasnegor, and H. Theakston, Lower back pain is reduced and range of motion increased after massage therapy. International Journal of Neuroscience, 2001. 106(3-4): p. 131-45.

109. Field, T., M. Hernandez-Reif, S. Seligman, and e. al., Juvenile rheumatoid arthritis:benefits from massage therapy. J Pediatr Psychol 1997. 22: p. 607-617.

110. Brattberg, G., Connective tissue massage in the treatment fo fibromyalgia. Eur J Pain, 1999. 3: p. 235-244.

111. Susnshine, W., T. Field, O. Quintin, and e. al., Fibromyalgia benefits from massage therapy and transcutaneous electrical stimulation. J Clin Rheumatol, 1996. 2: p. 18-22.

112. Field, T., M. Diego, M. Hernandez-Reif, and J. Shea, Hand arthritis pain is reduced by massage therapy. Journal of Bodywork and Movement Therapies 2007. 11(1): p. 21-24.

113. Field, T., M. Hernandez-Reif, M. Diego, S. Schanberg, and C. Kuhn, Cortisol decreases and serotonin and dopamine increase following massage therapy. International Journal of Neuroscience, 2005. 115(10): p. 1397-413.

114. Zeitlin, D., S. Keller, S. Shifflet, S. Schleifer, and J. Bartlett, Immunological effects of massage therapy during academic stress. Psychosomatic Medicine, 2000. 83-86(1).

115. Kuriyama, H., S. Watanabe, T. Nakaya, I. Shigemori, M. Kita, N. Yoshida, D. Masaki, T. Tadai, K. Ozasa, K. Fukui, and J. Imanishi, Immunological and Psychological Benefits of Aromatherapy Massage. Evid Based Complement Alternat Med, 2005. 2(2): p. 179-184.

116. Hernandez-Reif, M., T. Field, G. Ironson, J. Beutler, Y. Vera, J. Hurley, M.A. Fletcher, S. Schanberg, C. Kuhn, and M. Fraser, Natural killer cells and lymphocytes increase in women with breast cancer following massage therapy. International Journal of Neuroscience, 2005. 115(4): p. 495-510.
117. Ironson, G., T. Field, F. Scafidi, and e. al., Massage therapy is asociated wtih enhancement of the immune system's cytotoxic capacity. Intern J Neuroscience, 1996. 84: p. 205-217.
118. Diego, M.A., T. Field, M. Hernandez-Reif, K. Shaw, L. Friedman, and G. Ironson, HIV adolescents show improved immune function following massage therapy. Int J Neurosci, 2001. 106(1-2): p. 35-45.
119. Shor-Posner, G., M. Hernandez-Reif, M.J. Miguez, M. Fletcher, N. Quintero, J. Baez, E. Perez-Then, S. Soto, R. Mendoza, R. Castillo, and G. Zhang, Impact of a massage therapy clinical trial on immune status in young Dominican children infected with HIV-1. J Altern Complement Med, 2006. 12(6): p. 511-6.
120. Altman, R., E. Asch, D. Bloch, G. Bole, D. Borenstein, K. Brandt, W. Christy, T. Cooke, R. Greenwald, and M. Hochberg, et al. , Development of criteria for the classification and reporting of osteoarthritis: Classification of osteoarthritis of the knee. Diagnostic and Therapeutic Criteria Committee of the American Rheumatism Association. Arthritis Rheum, 1986. 29(8): p. 1039-1049.
121. Jacobs, J., A.L. Williams, C. Girard, V.Y. Njike, and D. Katz, Homeopathy for attention-deficit/hyperactivity disorder: a pilot randomized-controlled trial. Journal of Alternative & Complementary Medicine, 2005. 11(5): p. 799-806.
122. Williams, A., P. Selwyn, S. Molde, V. Yanchou-Njike, R. McCorkle, D. Zelterman, and D. Katz, A randomized controlled trial of meditation and massage effects on quality of life in people with advanced AIDS: a pilot study. JAIDS, 2004. Under Review.
123. Yale-Griffin Prevention Research Center, Understanding the determinants of and barriers to fruit and vegetable consumption in multi-ethnic adults. 10/01 - 9/03, Centers for Disease Control, Grant #U48-CCU115802, SIP 21-01.
124. Yale-Griffin Prevention Research Center, CORP Systematic Review (funded Yoga/Asthma, ADHD & Homeopathy, OA Massage & MCP studies). 10/00 - 1/04, Centers for Disease Control, Grant #U48-CCU115802.
125. Williams, A.L., A. Cotter, A. Sabina, C. Girard, J. Goodman, and D.L. Katz, The role for vitamin B-6 as treatment for depression: a systematic review. Fam Pract, 2005. 22(5): p. 532-7.
126. Williams, A.L., C. Girard, D. Jui, A. Sabina, and D.L. Katz, S-adenosylmethionine (SAMe) as treatment for depression: a systematic review. Clin Invest Med, 2005. 28(3): p. 132-9.
127. Williams, A.L., D. Katz, A. Ali, C. Girard, J. Goodman, and I. Bell, Do essential fatty acids have a role in the treatment of depression? J Affect Disord, 2006. 93(1-3): p. 117-23.
128. Ali, A. Research Training In Integrative Medicine: Ruth L. Kirschstein National Research Service Award (NRSA) Individual Postdoctoral Fellowship. NIH/NCCAM F32 AT002667. 2004 [cited 2008.
129. Association, A.M.T. American Massage Therapy Association. Glossary of terms. 2006 [cited 2007 Jan. 29].

130. Scaturo, D.J., The evolution of psychotherapy and the concept of manualization: An integrative perspective Pro Psychol Res Prac 2001. 5: p. 522-530.
131. Hart, T. Treatment definition in exeprience-based rehabilitation research. 2008 [cited 2008 June 17]; Available from: http://www.ncrrn.org.
132. Levin JS, Glass TA, Kushi LH, Schuck JR, Steele L, and J. WB., Quantitative methods in research on complementary and alternative medicine: A methodological manifesto. Med Care, 1997: p. 1079-1094.
133. Carter, B., Methodological issues and complementary therapies: Researching intangibles? Complem Ther Nurs Midwifery. , 2003. 9: p. 133-139.
134. Institute of Medicine of the National Academies, Need for innovative designs in research on CAM and conventional medicine in Complementary and Alternative Medicine in the United States. 2005. p. 108-128.
135. Schnyer, R. and J. Allen., Bridging the gap in complementary and alternative medicine research: Manualization as a means of promoting standardization and flexibility of treatment in clinical trials of acupuncture. J Altern Compl Med, 2002. 8(5): p. 623-634.
136. Bernstein, S., M. Laouri, L. Hilborne, and e. al., Coronary angiography: A literature review and ratings of appropriateness and necessity, RAND Report JRA-03 1992: Santa Monica (CA).
137. Jones, J. and D. Hunter, Qualitative research: Consensus methods for medical and health services research. BMJ 1995. 311: p. 376-380.
138. Anagnostis, C., T.G. Mayer, R.J. Gatchel, and T.J. Proctor, The million visual analog scale: its utility for predicting tertiary rehabilitation outcomes. Spine, 2003. 28(10): p. 1051-60.
139. Price, D.D., F.M. Bush, S. Long, and S.W. Harkins, A comparison of pain measurement characteristics of mechanical visual analogue and simple numerical rating scales. Pain, 1994. 56(2): p. 217-26.
140. Luttgens, K. and N. Hamilton, Kinesiology: Scientific Basis of Human Motion. 9th Ed. ed. 1997, Madison, WI: Brown & Benchmark.
141. Schafer, J.L., Analysis of Incomplete Multivariate Data. 1997, New York: Chapman and Hall.
142. Ezzo, J., What can be learned from Cochrane Systematic Reviews of massage that can guide future research? The Journal of Alternative and Complementary Medicine, 2007. 13(2): p. 291-295.
143. Hollis, S. and F. Campbell, What is meant by intention to treat analysis? Survey of published randomised controlled trials. BMJ, 1999. 319(7211): p. 670-4.
144. Sherman, K., C. Cherkin, J. Kahn, J. Erro, A. Hrbek, R. Deyo, and D. Eisenberg, A survey of training and practice patterns of massage therapists in two US states. BMC Complement Altern Med, 2005. 14(5): p. 13.
145. Katz, D., Clinical Epidemiology & Evidence-Based Medicine. 2001, Thousand Oaks: Sage Publications p. 169.
146. O'Leary, K.D. and T.D. Borkovec, Conceptual, methodological, and ethical problems of placebo groups in psychotherapy research. Am Psychol, 1978. 33(9): p. 821-30.

SUPPLEMENTS/APPENDICES

APPENDIX A: SCHEDULE OF EVENTS

	Weeks										
	0	1	2	3	4	5	6	7	8	16	24
Informed Consent	•										
Eligibility Determination	•										
Limited History	•								•	•	•
Demographic Data Collection	•										
Randomization	•										
Intervention (Massage or Wait-List Control)		•	•	•	•	•	•	•	•		
WOMAC Assessment	•								•	•	•
Visual Analog Pain Scale (VAS)	•								•	•	•
Range of Motion (ROM)	•								•	•	•
Timed walk of 50 ft	•								•	•	•
Daily Concomitant Medication and Adverse Event Log	•	•	•	•	•	•	•	•	•	•	•
